# Enzyme responsive antimicrobial hyaluronan-nanocellulose hybrid wound dressings for the treatment of infected wounds

**DOI:** 10.1016/j.bioactmat.2026.01.042

**Published:** 2026-02-11

**Authors:** Elisa Zattarin, Wasihun Bekele Kebede, Zeljana Sotra, Rozalin Shamasha, Annika Starkenberg, Valentina Guerrero-Florez, Lalit Pramod Khare, Torbjörn Bengtsson, Hazem Khalaf, Emma M. Björk, Jonathan Rakar, Johan P.E. Junker, Daniel Aili

**Affiliations:** a Laboratory of Molecular Materials, Division of Biophysics and Bioengineering, Department of Physics, Chemistry and Biology, Linköping University, SE-581 83, Linköping, Sweden; b Unit of Health Biotechnology, Institute of Biotechnology, Addis Ababa University, P.O. Box 1176, Addis Ababa, Ethiopia; c Center for Disaster Medicine and Traumatology, Department of Biomedical and Clinical Sciences, Linköping University, SE-581 85, Linköping, Sweden; d Division of Nanostructured Materials, Department of Physics, Chemistry and Biology (IFM), Linköping University, SE-58183, Linköping, Sweden; e Department of Microbiology, Immunology and Reproductive Science, School of Medical Sciences, Örebro University, SE-70362, Örebro, Sweden

**Keywords:** Wound infections, Wound dressing, Nanocellulose, Hyaluronic acid, Antimicrobial peptides

## Abstract

Wound infections pose a substantial clinical challenge and an escalating healthcare burden, further complicated by the rapid increase in multidrug-resistant bacteria. Antimicrobial peptides (AMPs) offer an alternative to conventional antibiotics, but their rapid degradation, hemolytic activity, and potential cytotoxicity complicate systemic delivery and can have negative impact on wound healing. Here we show a bacterial nanocellulose hyaluronan (BC-HA) hybrid hydrogel wound dressing functionalized with mesoporous silica nanoparticles (MSNs) for localized, enzyme responsive delivery of a sequence optimized antimicrobial peptide (SOAP) for treatment of infected wounds. The dressings provide moisture retention and excellent skin conformability while enabling infection-triggered AMP release by bacterial and host proteases. *In vitro*, SOAP-loaded dressings showed potent activity against clinical wound pathogens while remaining compatible with human primary dermal fibroblasts and keratinocytes. In a contaminated porcine wound model, the dressings significantly reduced bacterial load while accelerating wound re-epithelialization and epithelial maturation compared to the controls. By integrating a dual-function hydrogel that promotes healing and provides on-demand antimicrobial activity, critical limitations in the use of AMPs in wound care can be addressed, providing new possibilities to treat infected wounds.

## Highlights

• Bacterial nanocellulose is grafted with an enzyme-responsive hyaluronic acid hydrogel.
• Hyaluronic acid network degrades selectively in infected wounds environments.
• Antimicrobial peptides are loaded into mesoporous silica carriers inside the hydrogel.
• Infection-associated enzymes accelerate peptide release and hydrogel breakdown.
• Dressings lower bacterial burden and enhance re-epithelialization in infected wounds.

## Graphical abstract

**Figure:**
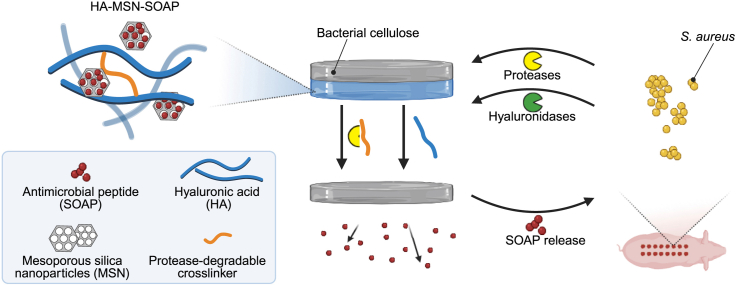

## 1 Introduction

Wound infections remain a significant global healthcare challenge that cause prolonged hospital stays, increased morbidity, and mortality, as well as a substantial economic burden on healthcare systems [1]. These infections are frequently caused by multidrug-resistant (MDR) pathogens, which thrive in the wound microenvironment, forming biofilms that resist conventional antimicrobial therapies [2]. Untreated wound infections can impede the healing process, promote wound chronification [3], and increase patient morbidity and mortality [4,5]. Current wound management strategies rely on administration of systemic antibiotics and topical antiseptics, wound debridement and cleansing of the wound, in combination with frequent dressing changes [6]. The efficacy of systemic antibiotics is, however, limited by poor penetration into biofilms [7], systemic side effects, and the increasing prevalence of antibiotic resistance [8]. In this context, innovative wound dressings with integrated, infection-responsive antimicrobial activity could both improve wound management and reduce reliance on systemic antibiotics, thereby addressing a critical gap in clinical wound care [9]. Topical delivery of antibiotics presents an attractive solution for enhancing efficacy while minimizing side effects [10]. Advanced wound dressings have been explored for localized topical antibiotic delivery based on drug eluting hydrogels, nanofibers, and bioactive polymers to achieve sustained and targeted drug release, while simultaneously supporting wound healing [[11], [12], [13], [14],^15^]. Stimuli-responsive dressings enable localized drug release in response to changes in the wound environment, such as variations in pH [16,17], temperature [18,19], enzymatic activity [20,21], excessive reactive oxygen species (ROS) production and elevated glucose levels [18], either individually or in combination [11], as well as by external stimuli, including phototriggering [[19], [20], [21]], and electrical stimulation [22]. Additionally, localized delivery strategies can enable the use of antimicrobial agents, such as antimicrobial peptides (AMPs) [23], that are currently unsuitable for systemic administration [24]. AMPs constitute a promising alternative to antibiotics for treatment of wound infections due to their broad-spectrum activity and unique mechanisms that minimize the development of resistance [25]. Derived from natural host defense systems, AMPs typically target microbial membranes, causing rapid disruption and cell death [26]. This mode of action makes AMPs effective against multidrug-resistant bacteria, fungi, and even biofilms [26,27]. In addition to their antimicrobial properties, many AMPs possess immunomodulatory functions, promoting wound healing by reducing inflammation and enhancing tissue regeneration [28,[29], [30]]. However, due to potential cytotoxicity and susceptibility to proteolytic degradation, topical delivery of AMPs must be tightly controlled [26]. Uncontrolled AMP release may lead to suboptimal antimicrobial activity and have a negative impact on wound healing [31]. Recent advancements in wound dressing-based AMP delivery systems have indicated possibilities to prevent AMP degradation, but often reduce the biocompatibility of the dressings resulting in impaired healing [32]. No current wound dressing integrates a clinically used dressing platform with dual enzyme-responsive AMP release and a healing-promoting hydrogel layer that addresses AMP toxicity, release control, and mechanical robustness in a single system.

**Fig. 1:**
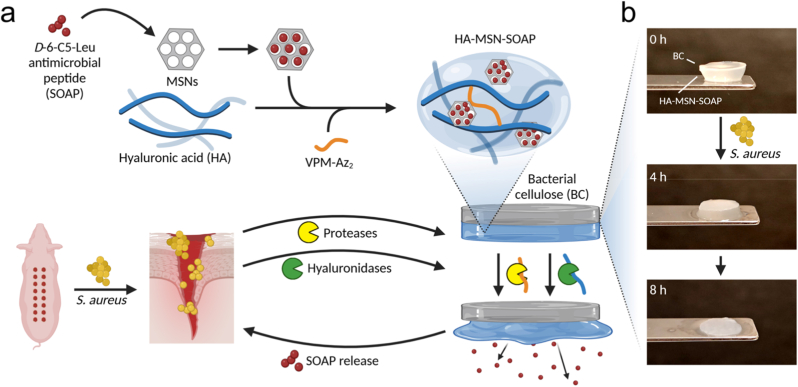
a) Schematic illustration of the design of the enzyme responsive antimicrobial BC-HA wound dressings for treatment of infected wounds. A protease and hyaluronidase responsive HA hydrogel with SOAP-loaded mesoporous silica nanoparticles (MSNs) was grafted to BC wound dressings. Degradation of the HA hydrogel results in release of SOAP and O-HA, reducing the infection level and promoting healing, while the BC remained intact to protect the wound. b) Photo of a BC-HA-MSN-SOAP wound dressing subjected to *S. aureus-*mediated degradation.

Here we present a clinically relevant bacterial nanocellulose–hyaluronan hybrid wound dressing that uniquely combines dual enzymatic responsiveness with controlled, high-capacity antimicrobial peptide delivery, enabling both effective infection suppression and promotion of early wound repair (Fig. 1). This work addresses key challenges in AMP delivery by integrating infection-responsive release, mitigation of AMP cytotoxicity, and preservation of clinically established dressing performance into a single system. Bacterial nanocellulose (BC) wound dressings can provide a moist and protected wound microenvironment and are used in the clinic for treatment of burns and hard-to-heal-wounds and as a cost-effective alternative to porcine xenografts [[33], [34]]. The high conformability and tissue mimetic nanostructure of the BC wound dressing used here (Epiprotect™) allow for close contact between the dressing and the wound, which prevents bleeding and reduces pain [32,33]. The nanoporous network formed by the entangled and randomly organized nanocellulose fibrils, makes the materials gas permeable while efficiently preventing bacterial contamination from the outside [35]. This nanostructure mimics the ECM, retains bioactive agents, maintains mechanical strength when hydrated, and allows transparency for direct wound monitoring [32,35,36]. However, while BC effectively prevents external contamination, it lacks intrinsic antimicrobial activity, and the moist environment beneath the dressing may facilitate proliferation of bacteria already present in the wound [35,37]. In non-infected wounds, the BC dressings can sit on the wounds for multiple weeks without the need for changes which is in stark contrast to other dressing materials that typically require regular and frequent changes [38]. In addition to high costs, pain, and low quality of life for patients, frequent dressing changes can have a negative effect on wound healing and expose the wound to pathogens [39]. To address this limitation, we functionalized BC dressings with an enzyme-responsive hyaluronic acid (HA) hydrogel for infection-triggered release of a sequence optimized antimicrobial peptide (SOAP), combining clinically established wound protection with controlled antimicrobial delivery and a HA-based strategy to counteract AMP toxicity and to contribute to rapid infection-free healing.

HA is a linear non-sulphated glycosaminoglycan (GAG) and one of the main components of the dermal and epidermal extracellular matrix (ECM). The role of HA in wound healing is still debated but HA has found wide application as a topical agent to promote wound healing in various forms, such as hydrogels, sponges, films or creams [40]. HA hydrogels have been shown to reduce scarring [41] and decrease healing time [42]. When applied on infected wounds, HA hydrogels can also reduce bacteria adhesion and biofilm formation [42,[43], [44]]. Moreover, the degradation of cross-linked low-molecular weight HA (LMW-HA) by hyaluronidases produces low molecular weight HA fragments (oligo-HA, O-HA) that can promote angiogenesis during the inflammatory phase of wound healing [45]. O-HA has also been seen to stimulate dermal fibroblast and keratinocyte migration and proliferation [45,46], and promote the formation of new extracellular matrix (ECM) [46], while maintaining a hydrated environment favourable for healing [47,48]. However, HA alone exhibits poor mechanical stability and chemical cross-linking or combination with other polymers is required to improve structural integrity and dressing performance [49]. By conjugating HA hydrogels to the BC wound dressings we overcome these limitations, while simultaneously mitigating AMP-associated cytotoxicity and enabling high-concentration AMP release with minimal impact on healing. Unlike previous BC dressings relying on conjugation or adsorption of AMPs with limited release control [50] and HA-based hydrogels where AMP release can compromise structural stability of the dressings [51], the present bilayer BC–HA system decouples wound protection, antimicrobial release, and healing support into a single, mechanically robust platform. The enzyme-responsive AMP-loaded HA hydrogel was cross-linked in situ and grafted onto Epiprotect™ BC dressings using a strain promoted azide-alkyne cycloaddition reaction with a protease cleavable peptide-based cross-linker. The resulting hybrid wound dressing combines the efficient wound protection properties of BC with infection-responsive antimicrobial activity and wound heling-promoting properties of HA, representing a significant step toward advanced bioactive wound dressings with translational potential. Strategies to load and tune the release of the sequence-optimized AMP D-6-C5-Leu (SOAP) [52] in the HA hydrogels were developed and optimized. SOAP was loaded either as peptide micelles or encapsulated in mesoporous silica nanoparticles (MSNs of SBA-15 type) [53]. SOAP is a 16 D-amino acid lipopeptide derived from the natural bacteriocin PLNC8 β with a proven efficacy against clinical isolates of the ESKAPE pathogens (*E. faecium*, *S. aureus*, *K. pneumoniae*, *A. baumannii*, *P. aeruginosa*, and *E. spp.*) and *E. coli* [52], which represent the most common wound pathogens [53,54]. SOAP is comprised entirely of D-amino acids and is thus resistant to proteolytic degradation [51,[55], [56]], making it an attractive AMP for generating antimicrobial protease-responsive wound dressings. The SOAP loaded dressings showed enzyme-triggered antimicrobial activity, excellent moist retention and skin conformability. The BC-HA-MSN-SOAP dressings effectively reduced bacterial load during early infection in an infected porcine wound model and supported wound re-epithelialization and epithelial maturation, demonstrating their ability to balance antimicrobial activity with tissue regeneration. This enzyme-responsive AMP delivery strategy thus represents a promising and translational approach to improve treatment of infected wounds and address the growing challenge of antibiotic resistance in wound care. Together, this design integrates infection-triggered antimicrobial action with HA-mediated healing support on a clinically used nanocellulose platform, directly addressing key translational barriers for AMP-based wound dressings.

## 2 Materials and methods

### 2.1 Materials

All materials were supplied by Merck Life Science AB (Stockholm, Sweden) unless otherwise specified and used as obtained. Bacterial cellulose (BC) wound dressings were supplied by S2Medical AB (Linköping, Sweden). BC membranes (ø 6 mm, and ∼400 μm thickness) were cut using a biopsy punch and stored in 70% ethanol and rinsed carefully in MQ water before use.

### 2.2 HA-BCN synthesis

Conjugation of *N*-[(1R,8S,9s)-Bicyclo [6.1.0]non-4-yn-9-ylmethyloxycarbonyl]-1,8-diamino-3,6-dioxaoctane (BCN) to hyaluronic acid (HA, M_w_ ∼100 kDa, Lifecore Biomedical Inc., Chaska, USA) was conducted as previously described [57]. Briefly, HA was dissolved in MES buffer (500 mg, 40 mL, 100 mм, pH 7). BCN (100 mg) was dissolved in 6 mL Acetonitrile (ACN): water (5:1 v/v) and mixed with HA and EDC (236 mg, 1.5 mmol) and HOBt (83 mg, 0.6 mmol) and the pH was adjusted to 7 and incubated for 24 h at room temperature under constant shaking. The product was precipitated in ice-cold ethanol, then dialyzed (Dialysis tubing, MWCO 4–6 kDa, Spectrum, Spectra/Por) twice against an aqueous 10% ACN solution and against water for seven days. The pH was adjusted to pH 6 and the product was diluted with milliQ water 1:1 before freeze drying. Purity and degree of modification was verified with ^1^H NMR in D_2_O using a 500 MHz NMR spectrometer (Bruker, Billerica, Massachusetts, United States). Data was processed with a Mnova software v. 12-03-21384 (Mestrelab Research, Santiago de Compostela, Spain).

### 2.3 Peptides synthesis

All peptides were synthesized by conventional Fmoc chemistry by microwave-assisted SPPS using a Liberty Blue Automated Microwave Peptide Synthesiser (CEM Corporation, *Charlotte, USA*). The peptide VPM-Az_2_ (N_3_-KGRDVPMS↓MRGGDRK-N_3_, where ↓ indicates the cleavage point) was synthesized as described previously [58]. Briefly, peptides were synthesized on a rink amide resin (0.19 mmol/g) using double coupling cycles of 3 min at 90 °C for all residues. Lys (Az) amino acid was used at the C- and N-terminal. The N-terminal amine was acetylated by reacting the peptide for 1 h in a solution of DMF: acetic anhydride 1:1 v/v. Cleavage from resin and deprotection was done using cleavage cocktail of TFA:H_2__O_:TIS:DODT (92.5:2.5:2.5:2.5 v/v/v/v) for 3 h. The SOAP peptide D-6-C5-N-Leu (D-[CH_3_-(CH_2_)_3_-CONH-LGLKLLWSAYKHRKTL]) with amidated C-terminal was synthesized on a rink amide resin (0.16 mmol/g) on a 0.1 mmol scale using 5-fold excess of reagents. Standard single coupling cycles of 3 min at 90 °C were employed, except for His residues where double coupling (50 °C, 10 min) was used. Activator N,N′-Diisopropylcarbodiimide DIC (5 eq) and activator base Oxyma (5 eq) were used, and Fmoc deprotection was achieved with Piperidine (20% v/v in DMF). A 5-C carbon tail (valeric acid) was conjugated at the C-terminal in the last step of the synthesis, by reaction with HBTU/DIPEA. A fraction of peptide was labelled with fluorophore 7-Amino-4-methyl-3-coumarinylacetic acid (AMCA) instead of the 5-C carbon tail, to enable spectroscopical tracking. The peptides were cleaved and globally deprotected with a TFA:H_2_O:TIS (95:2.5:2.5 v/v/v) cleavage cocktail for 2 h, and precipitated twice in cold diethyl ether. Peptide purification was performed on a C18 reverse phase column (ReproSil Gold 120C18, Dr. Maisch GmbH) attached to a semipreparative HPLC system (Dionex) using an aqueous gradient of acetonitrile (ACN) (10%-90%) containing 0.1% TFA. Peptide identity was confirmed with a MALDI-TOF Mass Spectrometer (UltrafleXtreme, Bruker Daltonics), with α-cyano-4-hydroxycinnamic acid matrix. SOAP aggregation was studied via dynamic light scattering (DLS) on a ALV/CGS-8F platform-based goniometer system equipped with a 632.8 nm HeNe laser (ALV-GmbH, Langen, Germany) with a collector oriented at 90° angle. SOAP stock solution (10 mм) was diluted with PBS buffer (10 mм, pH 7.4) to concentrations of 0.001–1000 μм. Samples were sonicated for 60 s and equilibrated at 37 °C for 10 min prior to the measurements. 10 runs of 30 s were acquired and manually averaged, and the data were analyzed with an ALV-Correlator software (3.0, ALV-GmbH, Langen, Germany).

### 2.4 Mesoporous silica nanoparticles (MSN, SBA-15) synthesis and characterization

SBA-15 MSNs were synthesized following a previously reported protocol [53]. A micelle solution was prepared by dissolving 2.4 g of Pluronic P123 (PEO_20_PPO_70_PEO_20_, $M_{w}∼$ 5800, Sigma-Aldrich) and 28 mg of ammonium fluoride (≥ 98%, Sigma-Aldrich) in 80 mL 1.84 M HCl (≥ 37%, Sigma-Aldrich) under magnetic stirring at 20 °C. A pre-mixed solution of 5.5 mL of tetraethyl orthosilicate (TEOS, 98%, Sigma-Aldrich) and 1.0 mL of heptane (99%, Sigma-Aldrich) was added to the micelle solution. The mixture was stirred for 4 min and kept under static conditions overnight at 20 °C and then transferred to a PTFE flask for hydrothermal treatment at 100 °C for 24 h. Finally, the material was calcined at 550 °C for 5 h (1 °C/min). The size, morphology, and pore structure of the as-prepared SBA-15 were determined using high-resolution transmission electron microscopy (HRTEM) and scanning electron microscopy (SEM). TEM was measured on the dispersion of the drop-cast particles onto a Formvar carbon-coated copper grid (Ted Pella), and images were taken with an FEI Technai G2 TF 20 UT microscope operated at 200 kV acceleration. For SEM, the as-prepared SBA-15 dry powder was dispersed on carbon tape and imaged using Zeiss LEO Gemini 1550 (Carl Zeiss, Oberkochen, Germany) with an acceleration voltage of 3 kV. The textual properties of the particles were characterized by Nitrogen-sorption measurements performed in an ASAP 2020 instrument (Micromeritics Instrument Inc., Norcross, GA, USA) operated at − 196 °C. Prior to measurement, the sample was outgassed overnight at 200 °C. The specific surface area was obtained using the Brunauer–Emmett–Teller (BET) equation [59], and the pore size distribution was determined using the Kruk–Jaroniec–Sayari (KJS) method [60] in the range of $P/P_{0}=0.07-0.18$.

### 2.5 Synthesis and grafting of HA±MSN±SOAP hydrogels to BC

Stock solutions of all hydrogel components were separately prepared. Detailed hydrogel composition and preparation procedure are summarized in Table S1, Supporting Information. Lyophilized HA-BCN was dissolved in PBS buffer (10 mм, pH 7.4) at a concentration of 30 mg/mL. VPM-Az_2_ stock solution was prepared in MQ water at a 20 mg/mL concentration. To prepare HA hydrogels, 11.67 μL of HA-BCN stock solution was cooled to 4 °C prior to being combined with 5.57 μL of MQ water, 0.56 μL of PBS stock solution (150 mм, pH 7.4) and 2.2 μL of VPM-Az_2_ stock solution to obtain a final volume of 20 μL. HA-SOAP hydrogels were prepared, by replacing 2 μL of MQ water with equal volume of SOAP stock solution (10 mм, prepared in MQ water). HA-MSN hydrogels were prepared by mixing 5.71 μL of MSN suspension (10 mg/mL, prepared in MQ water) with 0.56 μL of PBS stock solution (150 mм, pH 7.4), 11.67 μL of HA-BCN (30 mg/mL) and lastly with 2.2 μL of VPM-Az_2_ solution (20 mg/mL). HA-MSN-SOAP hydrogels were prepared by resuspending SBA-15 powder in MQ water (35 mg/mL) and dispersed by ultrasonication (10 min). 2 μL of SOAP stock solution (10 mм), was diluted with MQ water to a final SOAP concentration of 500 μм followed by addition of MSN stock solution (5.71 μL) and 10 min of sonication and 2 h incubation at RT to allow SOAP loading. MSN-SOAP suspension was then incubated at 37 °C and allowed to dry. The MSN-SOAP powder was then rehydrated with a pre-mixed solution of 0.56 μL of 150 mм PBS and 3.57 μL MQ water and thoroughly sonicated to disperse. 11.67 μL of HA-BCN were added to the dispersion and vortexed thoroughly, prior to being cooled to 4 °C and supplemented with 2.2 μL of VPM-Az_2_ stock solution (20 mg/mL). Hydrogel grafting to BC dressings was achieved by dispensing 20 μL of the mixed hydrogel components on the surface of hydrated 6 mm ø BC and allowing for the gelation to occur at 37 °C for 2 h in a humid environment.

### 2.6 SEM

The dressings were sectioned using a surgical blade and placed over conducting carbon tape before freeze-drying. The samples were sputtered with platinum for 10 s and imaged with a Zeiss LEO Gemini 1550 (Carl Zeiss, Oberkochen, Germany) with an InLens detector and an acceleration voltage of 3 kV. SEM images were processed with ImageJ2 Fiji software and false-colored using Gimp 2.10.38 software.

### 2.7 Transmittance measurements

Transmittance of the dressings was evaluated by measuring absorbance in the range 400–750 nm with a microplate reader (Tecan Infinite M1000 Pro, Tecan Austria GmbH, Grödig/Salzburg, Austria). Percent transmittance was evaluated with the formula:

$$\%T=100*10^{-A}$$

where A is the absorbance (n = 6).

### 2.8 Rheology

Rheological measurements were performed with a Discovery HR-2 rheometer (TA instruments, New Castle, DE, USA). A Peltier system was used to control temperature, and a 20 mm, 1° cone-plate geometry was employed. The instrument was equilibrated at 4 °C prior to starting the measurement and 45 μL of the gel was deposited onto the plate. The temperature was quickly raised to 37 °C prior to starting the experiment, and the oscillatory rheology measurement was carried on at 1% oscillation strain and 1 Hz frequency for 2 h. Gelation kinetics was monitored by evaluating Storage Modulus (G′), Loss Modulus (G″) and phase angle (°). Three replicates were averaged, and the mean and standard deviation were obtained. Following gelation measurement, amplitude sweep (1 Hz, 0.01–50%) was carried out, followed by a frequency sweep measurement (1%, 0.01–100 Hz). Strain recovery measurements were performed by subjecting BC-HA±MSN±SOAP dressings to alternating low- and high-amplitude oscillatory shear strain cycles. Low-strain intervals were applied at 1% strain within the linear viscoelastic region (LVR), while high-strain intervals were applied at 400% strain outside the LVR. A constant frequency of 1 Hz and temperature of 37 °C was maintained throughout the measurement, which was performed using a 8 mm plate geometry.

### 2.9 Hydrogel stability

BC-HA±SOAP, and BC-HA-MSN±SOAP dressings were prepared following the protocol described above, then incubated in 10 mм PBS, pH 7.4 at 4 °C for 16 months.

### 2.10 Hydrogel swelling

Swelling of dressings was evaluated in PBS. The dressings (BC, BC-HA±SOAP, and BC-HA-MSN±SOAP) were prepared following the protocol described above (*n* = 4). Following cross-linking, the dressings were weighted to determine initial weight ($W_{i}$) and subsequently submerged in 200 μL PBS buffer (10 mм, pH 7.4) and incubated at room temperature. At predetermined timepoints the buffer was removed, the dressings were tapped with wet tissue paper to remove excess liquid and weighted ($W_{t}$). Swelling was calculated as follows:

$$\%Swelling=\left(W_{t}-W_{i}\right)/W_{i}\;*100$$

### 2.11 Water retention ratio (WRR)

The dressings (BC, BC-HA±SOAP and BC-HA-MSN±SOAP) were prepared as described above (*n* = 4). The hydrogels were equilibrated in 200 μL PBS buffer (10 mм, pH 7.4) for 24 h, then wiped of excess water and placed on parafilm pieces. The initial wet weight ($W_{w}$) was recorded for each sample. The hydrogels were incubated at room temperature in an open-mouth container and allowed to dry. The samples' weight was measured every 30 min, and the weight at each timepoint t was recorded ($W_{t}$). The samples were subsequently incubated at 45 °C oven and the dry weight ($W_{d}$) was measured. WRR was calculated as:

$$\%WRR=\left(W_{t}-W_{d}\right)/\left(W_{w}-W_{d}\right)*100$$

### 2.12 SOAP release

Release of SOAP was investigated by incubating the dressings in 250 μL PBS buffer (10 mм, pH 7.4) or PBS buffer (10 mм, pH 7.4) supplemented with 5 mм CaCl_2_ and 0.5 mg/mL Collagenase Type-I (Coll-T1, from *Clostridium histolyticum,* ≥ 125 CDU/mg, Merck Life Science AB, Stockholm, Sweden) to evaluate degradation. The dressings were incubated in 2 mL Eppendorf tubes at 37 °C under constant shaking. At predetermined timepoints (0 h, 1 h, 2 h, 4 h, 6 h, 8 h, 24 h), SOAP release was measured with fluorescence spectroscopy (Fluoromax 4, Horiba scientific, with FluorEssence software) using λ_ex_ = 350 nm and λ_em_ = 450–750 nm. At each timepoint, peptide release was evaluated using two protocols corresponding to the conditions referred to as “non-centrifuged” and “centrifuged” throughout the manuscript. Protocol a) was used to determine the total concentration of released peptide, while protocol b) was used to determine the concentration of free peptide in the supernatant. Protocol a) involved homogenizing the supernatant by gentle pipetting, to resuspend any precipitate, and transferring of 100 μL of the same to a 96-well plate for reading. Protocol b) involved mixing the supernatant by gentle pipetting, then transferring of 200 μL of supernatant to a new Eppendorf tube, which was subsequently centrifuged (10 min, 10,000 rpm). 100 μL of the supernatant were then extracted and measured, before being returned to the second Eppendorf tube, vortexed and transferred back to the dressing-containing tube. Kinetic analysis was conducted using the average values ± standard deviation obtained from five independent experiments. The results are presented as the cumulative percentage of SOAP release over time calculated as:

$$\%Cumulative\;release=C_{t}/C_{i}*\;100$$

where $C_{t}$ indicates SOAP concentration at time t as determined by fluorescence measurements, and $C_{i}$ indicates the total releasable concentration of SOAP. To characterize the release behaviour of SOAP from BC-HA-MSN-SOAP dressings, three established kinetic models were evaluated as previously reported in the literature [61].

### 2.13 Dressing degradation by collagenase type I (Coll-T1)

BC-HA±MSN dressings were prepared as described previously and allowed to equilibrate overnight in 50 μL of PB buffer (10 mм, pH 7.4) supplemented with 5 mм CaCl_2_. Coll-T1 was dissolved in PB buffer (10 mм, pH 7.4) supplemented with 5 mм CaCl_2_ at a concentration of 0, 0.01, 0.05, 0.1 and 0.5 mg/mL and 1 mL of solution was supplemented to each dressing (*n* = 3). Incubation was carried out at 37 °C, and weight loss was recorded at 0 h, 1 h, 2 h, 4 h, 6 h, 8 h and 24 h.

### 2.14 Bacteria culture

*S. aureus* (strain ATCC 29213, MSSA, ATCC, Manassas, VA, USA) was kindly provided by the Torbjörn Bengtsson lab (Cardiovascular Research Centre, School of Medical Sciences, Örebro University, Sweden), and stored at −80 °C. Prior to use, a 10^9^ CFU/mL suspension was 1000-fold diluted in Mueller Hinton Broth (MHB, Millipore, Massachusetts, United States) and overnight cultured at a temperature of 37 °C. Mueller-Hinton agar plates (MHA) were used as solid support for CFU quantification.

### 2.15 *S. Aureus*-mediated dressing degradation

For investigation of *S. aureus*-mediated degradation of BC-HA and BC-HA-MSN dressings, *S. aureus* was cultured overnight in MHB to the stationary phase, then diluted to 10^5^ CFU/mL in either 1x PBS or MHB. Dressings swollen overnight in the respective buffers were incubated in 1 mL of (i) PBS at 37 °C, (ii) MHB at 21 °C and (iii) MHB at 37 °C. The dressings (*n* = 3) were incubated for a total of 24 h, and the weight of the dressings was monitored at 1 h, 2 h, 4 h, 6 h, 8 h and 24 h. Mass loss was normalized to the initial weight. In parallel, bacteria proliferation was evaluated for the same conditions by colony count, using MHA.

### 2.16 Microdilution assay

Sterile BC-HA-SOAP and BC-HA-MSN-SOAP dressings, along with suitable controls (SOAP solution and MSN-SOAP, with any excluded components substituted with 10 mм PBS buffer) were prepared in triplicate. Following a >2 h gelation time (37 °C, humid environment), the dressings were incubated in 250 μL of 10 mм PBS buffer (pH 7.4) and incubated for 6 h at 37 °C under shaking. Meanwhile, *S. aureus* culture was initiated by suspending 20 μL of 10^9^ CFU/mL *S. aureus* suspension in 20 mL MHB for overnight incubation (37 °C under gentle oscillation). The obtained bacteria culture was diluted to 10^6^ CFU/mL in MHB and 100 μL of the same was dispensed in a 96-well plate. Separately, the dressings were removed from the respective incubation solution, which was homogenized and 2-fold diluted for a total of 8 dilutions. 100 μL of such solutions were dispensed in the well plate, to lead to a 1:1 v/v bacteria: eluate solution. The plates were incubated for 20 h at 37 °C under shaking, after which MIC was determined spectroscopically by measuring optical density of the solution at 620 nm with an Epoch Microplate Spectrophotometer (BioTek, USA). The wells presenting inhibition were further plated on MHA (5 μL, *n* = 3) to evaluate bactericidal activity. The plates were incubated overnight, after which the colony growth was assessed.

### 2.17 Time-kill assay

BC-HA±MSN±SOAP dressings (*n* = 3) were incubated with 1 mL of a ∼10^5^ CFU/mL *S. aureus* at 37 °C for 24 h. Aliquots were extracted at 0 h, 2 h, 4 h, 6 h, 8 h and 24 h, 10-fold diluted with 10 mм PBS and plated on MHA plates for colony count. Data were plotted as average and standard error of the mean.

### 2.18 Primary cell isolation and culture

Human primary fibroblasts and keratinocytes were obtained from healthy patients undergoing routine abdominoplasty or mammoplasty at Linköping University Hospital, Linköping, Sweden under ethical permission (Swedish Ethical Review Authority no. 2018/97–31). Briefly, fibroblasts were isolated by mechanical dissection and enzymatic digestion of the dermis under sterile conditions. Skin samples were repeatedly washed in sterile PBS and subcutaneous fat mechanically removed. The remaining dermis was dissected into 1 × 3 mm^2^ pieces, placed in Dulbecco's modified Eagle's medium containing low glucose, L-Glutamine, Sodium pyruvate, phenol red (DMEM; Gibco Thermo Fisher Scientific) supplemented with 165 U mL^−1^ Collagenase (Gibco Thermo Fisher Scientific) and 2.5 mg/mL Dispase (Gibco Thermo Fisher Scientific) and incubated at 37 °C, 5% CO_2_, and 95% humidity for 24 h. After enzymatic digestion, the suspension was centrifuged for 5 min at 200 g and resuspended in DMEM containing 10% fetal calf serum (Gibco Thermo Fisher Scientific), 50 U/mL penicillin, and 50 mg/mL streptomycin. Cells were seeded into 75 cm^2^ culture flasks (Falcon, Corning Inc; Corning, NY) and incubated at 37 °C, 5% CO_2_, and 95% humidity. Medium was changed three times per week. Human primary keratinocytes were isolated by mechanical dissection and enzymatic digestion of the epidermis under sterile conditions. Skin samples were repeatedly washed in PBS. Subcutaneous fat was removed using scissors, cut into 3 × 6 mm pieces, placed in DMEM containing 25 U/mL Dispase and incubated at 4 °C for 18 h. The epidermis was manually removed from the dermis with forceps and minced with scissors. The minced epidermis was transferred to a 50 mL Falcon tube containing 4 mL 0.02% Versene (Gibco Thermo Fisher Scientific)/0.1% Trypsin (Gibco Thermo Fisher Scientific) 1:1. Tubes were incubated at 37 °C, 5% CO_2_ and 95% humidity for 15 min and vortexed every 2 min. Thereafter, 25 mL cell culture medium was added, and the tubes centrifuged at 365 g for 5 min. Supernatant was removed and the pellet resuspended and seeded in 75 cm^2^ culture flasks (Falcon, Corning Inc; Corning, NY) containing keratinocyte serum free medium with L-Glutamine (KSFM; Gibco Thermo Fisher Scientific, Paisley, UK). Medium was changed three times per week.

### 2.19 Cytocompatibility

Cytocompatibility of BC-HA-SOAP and BC-HA-MSN-SOAP dressings and relevant controls was evaluated on primary human keratinocytes and fibroblasts. Cells were detached using 0.02% versene and 0.1% trypsin and seeded in a 96-well plate at a concentration of 2000 cells/well and allowed to attach and grow for 24 h. Sterile dressings were incubated in 250 μL of 10 mм PBS for 6 h, after which the dressings were discarded. 10 μL of the eluate-rich solution was added to 135 μL of the fresh cell culture medium and supplemented to each cell-seeded well. Suitable controls were prepared by replacing any excluded component with 10 mм PBS buffer. Cell proliferation and migration speed were evaluated over a period of 72 h with a LiveCyte II kinetic cytometer (Phase Focus, Sheffield, UK). Data processing was performed with Prism 10 (GraphPad LLC, La Jolla, CA, USA) (*n* ≥ 5). Migration distance was measured with Excel and histograms were created with Prism (bin size: 2 for primary human keratinocytes, and 20 for primary human dermal fibroblasts) and fit with a Gaussian curve.

### 2.20 Porcine *in vivo* contaminated wound model

The antimicrobial efficacy of the dressing was evaluated using a porcine *in vivo* contaminated wound model. All described procedures were conducted under approval from the Regional Animal Ethics Committee (ID 1418) and adhered to the guidelines postulated by Linköping University. The study involved educated personnel and was supervised by a veterinarian. Two female pigs (*Sus scrofa domesticu*s) weighing 40–50 kg and aged 3–4 months were used in the study. Animals were acclimatized one week prior to the study; housed in boxes measuring 2 × 2.5 m at a state-of-the-art facility, with a light/dark circle of 12/12 h, an ambient temperature of 18–20 °C, daily feeding and free access to water and hay. General anaesthesia was induced by intramuscular (IM) injection of 10 μg/kg dexmedetomidine (Dexdomitor; Orion Pharma, Danderyd, Sweden) and 3 μg/kg of tiletamine and zolazepam (Zoletil; Virbac, Kolding, Denmark). Intubation was performed with an endotracheal tube connected to an automatic ventilator. Furthermore, the general anaesthesia and analgesia were maintained with intravenous infusion of 3–7.5 mg/kg pentobarbital sodium (Pentobarbitalnatrium vet.; APL, Kungens Kurva, Sweden) in combination with 0.5–0.75 μg/kg fentanyl (Leptanal, Janssen, Solna, Sweden). Vital parameters were monitored by pulse oximetry, capnography and rectal thermometer. Signs of postoperative pain were treated with IM administration of 50–75 μg fentanyl and 40 mg meloxicam (Loxicom; N-Vet AB, Uppsala, Sweden). Firstly, dorsal and lateral hair was removed with sugar wax (Veet, Kanata, Canada), followed by antiseptic preparation of skin with three repetitions of 70% ethanol and iodine application. The skin was thereafter contaminated with 10^4^ CFU/mL *S. aureus* (ATCC 29213). 20 partial-thickness wounds per patient, were created paravertebrally using 10 mm biopsy punches (Acu-Punch; Acuderm Inc, Fort Lauderdale, USA). Four conditions were evaluated: BC-HA, BC-HA-SOAP, BC-HA-MSN-SOAP and a commercially available foam dressing (Sorbact® Foam, Essity, Hamburg, Germany). Sterile dressings (ø 25 mm) were positioned onto the wounds on day 0. On day 2 and 4, the old dressings were carefully removed, and photographs of the wounds were acquired. Then, new dressings were positioned onto the wounds and covered with squared 25 × 25 mm Leukotape Foam (Essity), to prevent unwanted dressing migration. Wounds were further sealed with one or two layers of Tegaderm™ Film (3M™, Solna, Sweden) and an elastic self-adhesive dressing (Hansbo Sport, Båstad Sweden). Samples were obtained on days 2, 4 and 7. Wounds were divided into two halves; one allocated to histology and one to quantitative bacterial cultures. The histology samples were dissected with a scalpel and included healthy skin margins. On the wound half assigned for quantitative cultures, three biopsies were obtained using 3 mm punches (Paramount Surgimed Ltd, Delhi, India). At the end of the study, the animals were euthanized by intravenous injection of 400 mg/kg pentobarbital sodium (Pentobarbitalnatrium vet.; APL, Kungens Kurva, Sweden) under general anaesthesia.

### 2.21 Histological analysis of porcine wounds

One biopsy per wound and timepoint was fixed in 4% neutral buffered paraformaldehyde (Histolab Products AB, Gothenburg, Sweden) for 16 h, dehydrated through an ethanol–xylene series and embedded in paraffin. A microtome (RM2255, Leica Biosystems, Wetzlar, Germany) was used to section the samples into 6 μm sections mounted on slides (Epredia Superfrost Plus Adhesion Microscope Slides; Gerhard Menzel GmbH, Braunschweig, Germany). Staining was performed using Hematoxylin and Eosin (Histolab Products AB) according to manufacturer's instructions. Coverslips (Gerhard Menzel GmbH, Thermo Fisher Scientific) were mounted on the stained slides using Pertex mounting medium (Histolab Products AB). Visualization was performed using a BX41 microscope (Olympus, Stockholm, Sweden) and images captured with a DP70 CCD camera (Olympus).

### 2.22 Immunofluorescent staining of porcine wounds

Selected tissue sections were used for immunofluorescent staining against *S. aureus* and PCNA*.* Briefly, sections were rehydrated through a xylene-ethanol series and hydrophobic barriers were created with a Liquid Blocker Super Pap-pen (Histolab Products AB, Stockholm, Sweden). Thereafter followed an incubation with blocking serum at room temperature in a humid chamber. Samples were incubated with primary antibodies (*S. aureus:* dilution 1:800, CAT #PA1-7246; Invitrogen, Thermo Fisher Scientific, Stockholm, Sweden, PCNA: dilution 1:100, NB500-106, Novus Biologicals, Stockholm, Sweden) or PBS (for negative controls), at 4 °C overnight in a dark humid chamber. Subsequently, the sections were incubated with a secondary anti-rabbit antibody (1:250, CAT #A11034, Invitrogen, Thermo Fisher Scientific) and 5% DAPI nucleic stain (CAT #D1306; Invitrogen, Thermo Fisher Scientific) for 1 h. Coverslips (Gerhard Menzel GmbH, Thermo Fisher Scientific) were mounted on the stained slides using Prolong Glass Antifade Mountant (Thermo Fisher Scientific). Samples were visualized using a Leica Stellaris 5 confocal microscope (Leica Microsystems AB, Wetzlar, Germany) with the LAS X microscope software (Leica Microsystems AB). Bacteria present in the dermis at day 7 were quantified using QuPath 0.6.0-rc3 software [62]. Dermis was manually outlined, and threshold settings adjusted to filter specific positive labeling of bacteria. The area of positive labeling was then divided by total area of the dermis for each sample. To quantify the fluorescence images of anti-*S. aureus*, all pixels over the threshold for positive signal in the 488-channel acquisition were counted and provided as a fraction of total annotated dermal area (excluding surface and epidermis due to wound variability). Four images from different samples per group were counted using the same technique in QuPath v0.6, and the results summarized and graphed in GraphPad Prism v9. Group statistics were compared using ANOVA with Holm-Sidák post-test, with adjusted p < 0.05 considered different.

### 2.23 Quantitative cultures of porcine wounds

Three biopsies per wound and timepoint were used for quantitative cultures. Biopsies were stored at −20 °C and thawed on the day of culture. Thawed samples were weighted, placed in RB sample tubes (Cat #990381; Qiagen) containing PBS, 5 mm stainless steel beads (Cat #69989; Qiagen) and subsequently homogenized using a TissueLyser (Qiagen, Sollentuna, Sweden). A dilution series of each sample was cultured in triplicates on Chapman agar. Plates were incubated overnight at 37 °C and bacterial colonies manually enumerated. CFU/g was generated through the mean value from the triplicate of each culture, multiplied with the dilution factor of the triplicate and divided by the biopsy weight.

### 2.24 Statistical analysis

Statistical analysis was performed using Prism (v. 10, GraphPad LLC, La Jolla, CA, USA). Data were analyzed using a one-way ANOVA test complemented with a suitable post-hoc test. Dunnett's multiple comparison test was used to compare each experimental condition against the control, while Tukey's multiple comparison test was chosen to compare each experimental condition to each other. Statistical significance was indicated as: ∗ (p < 0.1), ∗∗ (p < 0.01), ∗∗∗ (p < 0.001) and ∗∗∗∗ (p < 0.0001).

## 3 Results and discussion

### 3.1 Hydrogel design

**Fig. 2:**
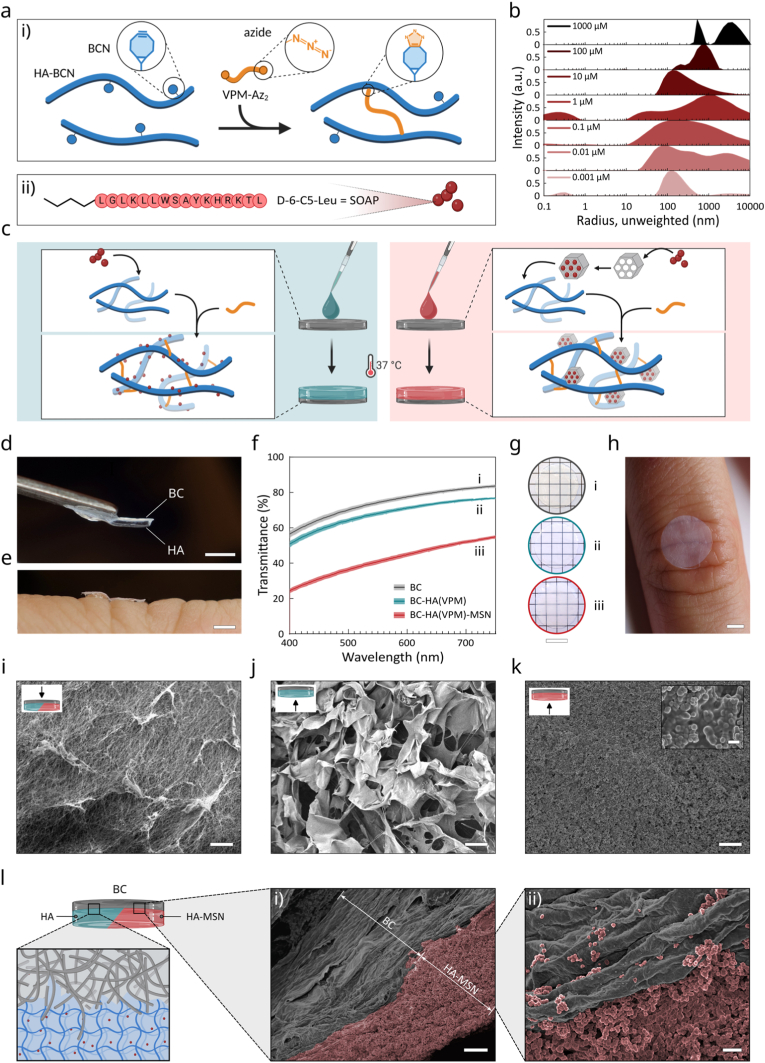
a) Schematic representation of i) the formation of HA hydrogels, ii) amino acid sequence of the sequence optimized antimicrobial peptide D-6-C5-Leu (SOAP). b) DLS measurement of SOAP peptide (0.001 μм – 1 mм) indicating the hydrodynamic radius. c) Schematic representation of BC-HA-SOAP (left) and BC-HA-MSN-SOAP dressings (right). d,e) Photograph of BC-HA wound dressing showing d) side view (scale bar: 2 mm), e) conformability over skin folds (scale bar: 2 mm). f) Transmittance spectra and g) photographs of circular (Ø 20 mm) i) BC, ii) BC-HA and iii) BC-HA-MSN hydrogels, scale bar: 10 mm. h) Transparency of BC-HA hydrogel on healthy skin (scale bar: 2 mm). i-l) Scanning electron micrographs (SEM) of BC-HA and BC-HA-MSN bilayer dressing, showing the i) BC-side (scale bar: 20 μm), j) HA-side (scale bar: 20 μm), k) HA-MSN side (scale bar: 20 μm. Inset: scale bar: 1 μm). l) Schematics of the bilayer hydrogel BC-HA-MSN microstructure and false-colored scanning electron micrographs of BC-HA-MSN cross-section at different magnifications (scale bar: i) 20 μm and ii) 2 μm).

To create an enzyme responsive and reservoir for release of AMPs (D-6-C5-Leu, SOAP) and a healing-promoting and biocompatible wound/material interface, we grafted a HA-based hydrogel with and without MSNs on the surface of Epiprotect™ BC wound dressings as schematically illustrated in Fig. 2. The HA hydrogels were obtained by the functionalization of hyaluronic acid (M_w_ ∼100 kDa) with bicyclononyne (BCN) using carbodiimide chemistry (Fig. 2a). The degree of substitution was approximately 14% (Fig. S1, Supporting Information). The HA-BCN was then combined with either SOAP micelles (Fig. 2b, Fig. S2, Supporting Information) or SOAP loaded in MSNs followed by addition of a protease-degradable cross-linker (VPM-Az_2,_ Fig. S3, Supporting Information) to initiate hydrogel formation (Fig. 2c). The copper-free strain-promoted azide-alkyne cycloaddition (SPAAC) cross-linking reaction is highly temperature dependent and proceeds very slowly at 4 °C [[63], [64], [65], [66]]. Following thorough mixing of the components at 4 °C, the hydrogel precursor was deposited onto bacterial cellulose wound dressings and the temperature was raised to 37 °C to induce cross-linking. By controlling the cross-linking kinetics with the temperature, we could ensure that some of the hydrogel components had time to diffuse into the uppermost layers of the fibrillar nanocellulose BC network, facilitating physical interlocking of the hydrogel in the BC network while preventing complete impregnation of the BC. During the cross-linking process, the HA hydrogel formed an interpenetrating network at the BC interface that firmly anchored the hydrogel to the surface of the dressings, resulting in a BC-HA hybrid material with a distinct bilayer appearance (Fig. 2d, Fig. S4, Supporting Information).

The thickness of the grafted hydrogel was tailored by the volume of hydrogel components. Addition of 20 μL of the hydrogel components to a ø 6 mm BC dressing resulted in a thickness of the grafted HA hydrogel of about 700 μm and a total dressing thickness of 1.1 mm. The addition of the soft HA hydrogel did not have any major impact on the mechanical flexibility of the dressings, and the materials were found to conform well to the irregularities of intact skin (Fig. 2e). The transparency of the BC dressings was largely retained after grafting, which can facilitate ocular inspection of a wound without the need for removal of the dressing (Fig. 2f–h). Including MSNs in the hydrogels, however, resulted in a slight drop in transmittance in the visible wavelength range due to the scattering by the particles, but the dressings were still relatively transparent. Scanning electron microscopy (SEM) micrographs confirmed the distinct topographies of the two sides of the BC-HA dressings (Fig. 2i–l, Fig. S5, Supporting Information). The BC side of the dressings retained its nanofibrillar structure, whereas the side with the grafted HA hydrogel had a more lamellar morphology, typical for dehydrated HA hydrogels under SEM [65]. MSNs included in the grafted HA hydrogel were homogeneously distributed and embedded in the hydrogel (Fig. S6, Supporting Information). The SEM images clearly demonstrated the vast number of MSNs that could be trapped in the BC-grafted HA hydrogel, indicating the potential to load high concentrations of AMPs in the BC-HA-MSN dressings.

### 3.2 Loading of SOAP in BC-HA and BC-HA-MSN dressings

**Fig. 3:**
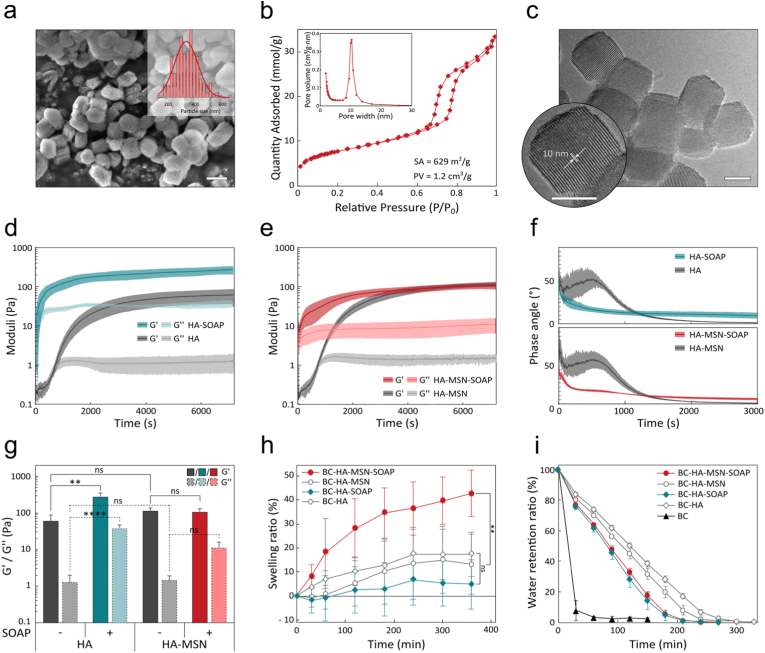
a) SEM micrographs of MSNs. Scale bar 400 nm. Inset: particle size distribution. b) Brunauer-Emmett-Teller (BET) nitrogen adsorption/desorption isotherms plot. Specific surface area (SA) and total pore volume (PV) are indicated. Inset: Pore size distribution of MSN measured using the KJS method. c) HRTEM micrograph of MSNs. Inset: TEM micrograph of MSNs. Scale bar 200 nm. d-e) Gelation kinetics of d) HA±SOAP and e) HA-MSN±SOAP hydrogels at 37 °C for 2 h (*n* = 3). f) Phase angle corresponding to the first 3000 s of the gelation time and g) Storage and loss moduli of the same hydrogels following a 2 h gelation (*n* = 3) at 1 Hz, 1% strain. Statistical analysis was conducted with an ordinary one-way ANOVA complemented with a Tukey's multiple comparison test on Storage moduli (continuous line) and on Loss moduli (dashed line). h) Swelling ratio (*n* = 4) and i) water retention ratio (*n* = 4) of BC, BC-HA±SOAP and BC-HA-MSN±SOAP dressings.

SOAP (D-6-C5-Leu) is a rationally designed antimicrobial lipopeptide derived from plantaricin NC8β with demonstrated broad-spectrum activity against Gram-positive and Gram-negative bacteria, including ESKAPE clinical isolates [52]. Its antibacterial action is primarily driven by rapid membrane permeabilization, with reported selectivity toward bacterial membrane mimics and low hemolytic activity *in vitro*. SOAP was included in the grafted HA hydrogels either as micelles or loaded in MSNs to be able to tune the concentration, bioavailability, and release kinetics of the peptides. Trapping of SOAP micelles was done by addition of 2 μL of a 10 mм SOAP stock solution to the HA hydrogel components prior to addition of the cross-linker, resulting in a final concentration of 1 mм in the grafted HA hydrogel. (Table S1, Supporting Information). Because of the amphipathic properties of the peptide, the SOAP assembled into colloidally stable micelles with a hydrodynamic radius (R_H_) greater than 370 nm in aqueous buffers at concentrations above the critical micelle concentration (CMC, 1.5 μм) (Fig. 2b, Fig. S7, Supporting Information). The mesh size of the cross-linked HA-based hydrogels is about 30–60 nm [67], which allows for rapid diffusion of individual peptides. SOAP micelles were larger than the hydrogel mesh size and were efficiently trapped in the HA hydrogel above the CMC, generating BC-HA-SOAP dressings. Likewise, the MSNs with a size of about 400 nm (Fig. 3a) were efficiently retained in the grafted HA hydrogels. The MSNs had a specific surface area of 629 m^2^^/^g and a pore size distribution of ∼10 nm (Fig. 3b and c), allowing for loading of large quantities of peptides. SOAP loading in MSNs was performed by incubating a suspension of MSNs with an aqueous solution of the peptide at pH ∼3. At this pH value, the positive net charge of the peptides (+5) resulted in favourable electrostatic interactions with the negatively charged MSNs. By then allowing the aqueous suspension media to slowly dry out, the concentration gradient and the capillary effect forced the peptides to enter the pores of the MSNs [61]. The resulting dried SOAP-loaded MSNs (MSN-SOAP) were collected as a dry powder. The SOAP-loaded MSNs were resuspended in PBS buffer prior to mixing with the hydrogel components and fabrication of the BC-HA-MSN-SOAP dressings (Table S2, Supporting Information).

The HA hydrogels were quite soft with a storage modulus (G′) of 62.2 ± 25.6 Pa and a gelation time of about 10 min as indicated by moduli crossover point (Fig. 3d–f). Including SOAP micelles at a final SOAP concentration of 1 mм in the HA hydrogels resulted in statistically significant increase in storage modulus to G’ = 284.5 ± 66.5 Pa and decrease in gelation time to ∼30 s (Fig. 3d–f). The increase in stiffness of the SOAP-loaded hydrogel could be attributed to the formation of electrostatic interactions between the SOAP micelles and the HA hydrogel backbone resulting in supramolecular cross-linking in addition to the covalent SPAAC cross-linking [68]. The supramolecular SOAP-mediated HA cross-linking occurred rapidly and could be identified as an immediate drop in the phase angle and increase in loss modulus (G″) from 1.3 Pa to 38.8 Pa (Fig. 3f,g, and Fig. S8, Supporting Information). The physical trapping of MSNs in the HA hydrogels did not result in any significant changes in viscoelastic properties compared to the HA hydrogels, with G’ = 117.8 ± 18.2 Pa and a gelation time of 723 s (Fig. 3e and f), despite a 51% increase in hydrogel dry weight from 19.7 mg/mL for the HA hydrogels to 29.7 mg/mL for HA-MSN hydrogels.

In contrast to SOAP micelles, the loading of SOAP in the MSNs prior trapping in the HA hydrogels did not induce any significant changes in stiffness with a G' = 108.8 ± 21.2 Pa (Fig. 3g), confirming that the SOAP peptides were largely retained in the MSNs. However, G'' increased to 11.3 Pa and the gelation was faster than for HA-MSN hydrogels with a rapid drop in phase angle, indicating some additional supramolecular cross-linking, which most likely was caused by SOAP peptides adsorbed on the surface of the MSNs contributing to electrostatic interactions with the HA polymer network. Overall, the loading of SOAP in the BC-HA±MSN dressings had a small but positive impact on the viscoelastic properties of the grafted HA-hydrogel, rendering them slightly more robust while retaining excellent conformability to the skin. The robustness of the dressings was further confirmed with cyclic strain recovery measurements, where the hydrogels were subjected to alternating low (1%) and high (400%) oscillatory strain (Fig. S9, Supporting Information). All formulations exhibited a pronounced decrease in G′ under high strain, followed by rapid and full recovery during low-strain intervals, indicating that the hydrogel network can accommodate large deformations without permanent damage or loss of stiffness. Interestingly, BC-HA-MSN and BC-HA-MSN-SOAP displayed the largest G′ drops, suggesting greater network deformation. The elevated G″ during high-strain intervals reflects energy dissipation, possibly associated with supramolecular interactions between MSN nanoparticles. The full recovery after release of the strain demonstrates that these hydrogels can withstand severe deformation while maintaining structural integrity, a critical feature for wound dressing applications. However, the overall mechanical behaviour of the composite BC-HA±MSN±SOAP dressing is mainly governed by the undegradable BC layer, which constitutes the stiffest component of the dressing. The BC material used in this study, Epiprotect™, has been previously characterized in terms of mechanical properties, and provides excellent stability and flexibility for wound dressing applications [69]. The dressings were structurally and chemically very robust and no apparent changes were observed over a period of 16 months (Fig. S10, Supporting Information). Due to the high concentrations of the broad-spectrum antimicrobial SOAP peptides in the dressings, maintaining sterility during storage was not an issue.

Wound dressings that retain moisture can stimulate wound healing by promoting faster re-epithelialization [70], and reduce scarring [71]. Moreover, moist and conformable wound dressings can reduce pain, which tends to reduce patient discomfort [72]. However, excess wound exudate must be removed to prevent tissue maceration [73]. The possibility to absorb moisture was determined by measuring the swelling ratio of the as-prepared hydrated dressing by immersing them in PBS for a period of 6 h (Fig. 3h). BC-HA-MSN-SOAP hydrogels displayed a swelling of 42.6% ± 9.5% after 6 h which can be attributed to the increased charge density arising from SOAP retention within the matrix, promoting osmotic influx of liquid from the surrounding medium. The BC-HA, BC-HA-SOAP, and BC-HA-MSN dressings showed a more moderate swelling, in the range of 5.0%–17.4% after 4 h, with no additional swelling after this. The dressings were thus almost fully swollen after fabrication but were found to adsorb some additional liquid. The similar swelling behaviour of BC-HA and BC-HA-SOAP indicates that SOAP was rapidly released when fully hydrated, which was further confirmed by the release experiments described in more detail below (Fig. 5). The water retention ratio (WRR) of the hydrogels was determined at room temperature and a relative humidity of 32%. The hydrogels were equilibrated in PBS for 24 h prior to the test. The native BC dressings showed the lowest WRR, since it was the thinnest of the materials (d∼400 μm), and was fully dry within 60 min (Fig. 3i). Grafting of the HA hydrogel to BC significantly improved WRR and the BC-HA and BC-HA-MSN dressings remained moist for up to 5 h and 4 h, respectively. SOAP loading resulted in a moderate decrease in WRR, resulting in complete drying within 3.5 h.

### 3.3 Hydrogel degradation

**Fig. 4:**
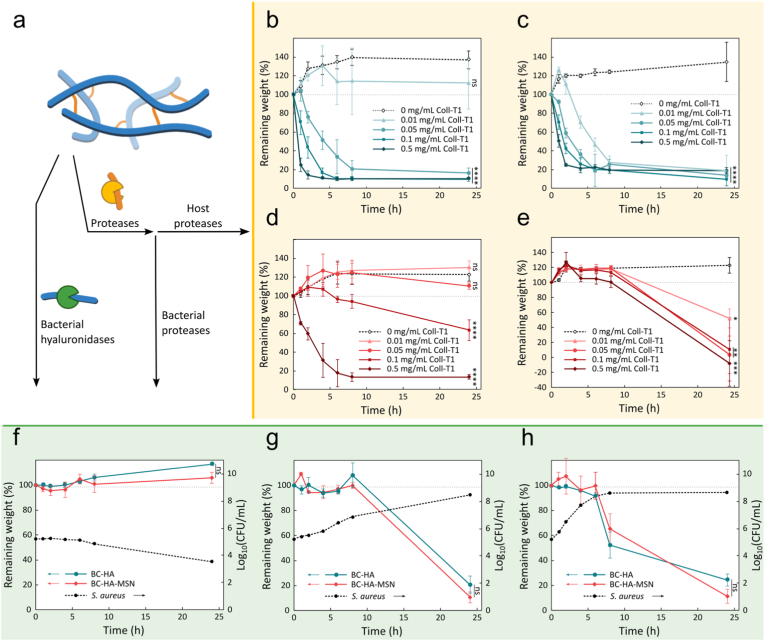
a) Schematic illustration of the degradation of HA hydrogels by host and bacterial proteases and hyaluronidases. b-e) Degradation of b) BC-HA, c) BC-HA-SOAP, d) BC-HA-MSN and e) BC-HA-MSN-SOAP hydrogels by 0–0.5 mg/mL Coll-T1. Statistical analysis was performed with ordinary one-way ANOVA corrected for multiple comparisons using Dunnett's test (*n* = 3). f-h) BC-HA and BC-HA-MSN hydrogel degradation by *S. aureus* bacteria, obtained by incubating a 6 mm Ø hydrogel in 1 mL of f) in PBS (37 °C), g) MHB (20 °C), h) MHB (37 °C). Statistical analysis was performed with ordinary one-way ANOVA corrected for multiple comparisons using Tukey test (*n* = 3).

While BC is resistant to hydrolysis by human enzymes [73,[74], [75]], the VPM-Az_2_ cross-linker can be cleaved by multiple proteases, including matrix metalloproteinases (MMPs) and bacterial proteases [[76], [77], [78], [79]]. Moreover, HA is readily degraded by hyaluronidases that can be expressed by both bacteria and human cells [[80], [81]], resulting in hydrogels that can be both degraded by wound pathogens and enzymes upregulated as a result of the infection-related inflammatory response of the host (Fig. 4a) [[64], [82]]. Protease degradation was explored by subjecting the dressings to increasing concentrations of collagenase type 1 (Coll-T1, 0–0.5 mg/mL) [77,78] and measuring mass loss over time. The collagenase concentrations used (0.01–0.5 mg/mL) were selected to reflect the physiological range of proteolytic activity in wounds, from low MMP levels in non-infected acute wounds to the 10–100-fold higher activities observed during infection and inflammation [[83], [84]]. Consistent with this, HA/VPM hydrogels remained intact at low enzyme concentrations but degraded rapidly at elevated levels, while the BC layer remained stable across all conditions. Both BC-HA and BC-HA-MSN dressings displayed a small gain in mass at low Coll-T1 concentrations (<0.05 mg/mL), as a result of the hydrogel swelling (Fig. 4b–d) [58]. At higher Coll-T1 concentrations (0.05–0.5 mg/mL), the grafted HA hydrogel was degraded rapidly, resulting in almost complete mass loss in 8 h. The degradation of the BC-HA-MSN dressings was slower and required higher Coll-T1 concentrations for complete degradation (0.5 mg/mL), likely due to the steric hindrance by the MSNs that could hamper Coll-T1 diffusion in the HA hydrogels. BC-HA-SOAP hydrogels exhibited degradation kinetics comparable to BC-HA, which is likely due to the rapid release of the SOAP peptide from the hydrogel. In contrast, BC-HA-MSN-SOAP hydrogels displayed slower degradation kinetics than their SOAP-free counterparts, likely due to the higher concentration and prolonged retention of the SOAP peptide within the hydrogel, which facilitates supramolecular cross-linking with the HA polymer network and sterically impedes Coll-T1 penetration.

**Fig. 5:**
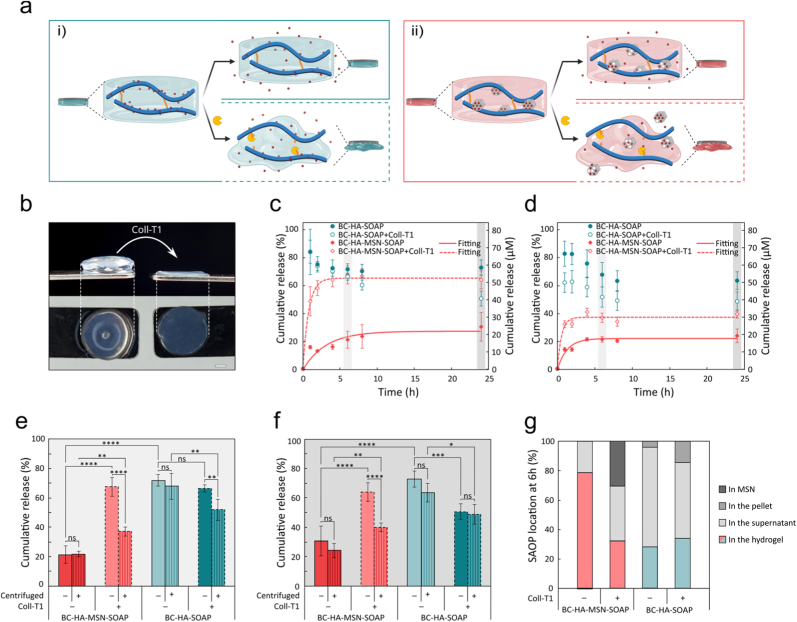
a) Schematic illustration of *in vitro* peptide release from i) BC-HA and ii) BC-HA-MSN dressings ± Coll-T1 illustrating SOAP spontaneous release by diffusion (continuous line) and by protease-triggered degradation (dotted line). b) Photo of BC-HA-SOAP dressing before and after addition of Coll-T1 (0.5 mg/mL, 24 h, 37 °C). Scale bar 1 mm. c-d) Peptide release from BC-HA-SOAP and BC-HA-MSN-SOAP dressings incubated ± Coll-T1 c) not subjected to centrifugation and d) subjected to centrifugation. Peptide release kinetics fitting shown when applicable using a first-order model. e-f) Cumulative SOAP release at e) 6 h and f) 24 h of incubation. Statistical analysis was performed with ordinary one-way ANOVA corrected for multiple comparisons using Tukey test (*n* = 5). g) SOAP localization after 6 h incubation ± Coll-T1.

In addition to proteases, the grafted HA hydrogels can be hydrolysed by both endogenous and bacterial hyaluronidases. Hyaluronidases secreted by *S. aureus* (HysA) are believed to play an important role in tissue colonization in infected wounds [85]. HysA cleaves the β-1,4 glycosidic bond in HA to generate nutrients and to promote bacteria dissemination and establish *S. aureus* biofilms [85]. The degradation of the grafted HA hydrogels by *S. aureus* (ATCC 29213) was evaluated by subjecting the hydrogels to three different bacteria colonization scenarios (Fig. 4f–h). For all three scenarios, the dressings were exposed to a concentration of *S. aureus* close to the 10^5^ CFU/mL clinical infection threshold [[86], [87]] at t = 0 h and then incubated with the bacteria for 24 h while the weight loss of the dressings was recorded. In the first scenario, the dressings were incubated in nutrient-free medium (PBS buffer, 10 mм) at 37 °C to maintain the metabolic activity of the bacteria while impeding bacterial proliferation. Hydrogel weight increased linearly with about 17% over the 24 h incubation, indicating a limited hydrogel degradation that resulted in additional swelling (Fig. 4f). In the second scenario, the dressings were incubated at suboptimal temperature for bacterial growth (21 °C) in a nutrient-rich medium (MHB) to reduce bacteria proliferation rate. During the first 8 h the bacteria count increased to about 6.9 log_10_ CFU/mL while the weight of the dressings remained nearly constant, likely due to a balance between swelling and degradation (Fig. 4g). Incubation for an additional 16 h resulted in nearly complete hydrogel degradation while the bacteria growth reached the stationary phase (8.5 log_10_ CFU/mL). The third scenario was set up to simulate an untreated wound infection where bacteria growth was promoted by a nutrient-rich medium and an optimal incubation temperature, allowing for *S. aureus* to reach the stationary phase within 6 h (Fig. 4h). Under these conditions, the degradation of the grafted HA hydrogels was rapid, with significant mass loss for both BC-HA and BC-HA-MSN dressings after 6 h. These results confirmed that a relevant wound pathogen could degrade the grafted HA hydrogels, which can be used for triggering release of encapsulated SOAP. Importantly, the nanocellulose was not affected, leaving the BC dressing intact to protect the wound (Fig. 5a and b).

To monitor the SOAP release, we replaced the C5 alkane tail in 25% of the SOAP peptides with a fluorescent aminomethylcoumarin acetate (AMCA) moiety (Fig. S11, Supporting Information). The release of SOAP encapsulated as micelles in the BC-HA dressings was rapid with 84.1 ± 8.2% release within 1 h, due to the rapid shift in equilibrium below the CMC of the peptide, while only a small fraction of peptide was retained due to electrostatic interactions with the HA backbone (Fig. 5c). Following the initial burst release, SOAP was to some extent re-adsorbed to the BC, leading to a total cumulative release of 66.5 ± 5.1% after 24 h (Fig. 5c–g). The re-adsorption is likely caused by interactions between SOAP and the BC [50]. Release of SOAP encapsulated in MSNs was significantly slower with 16.0 ± 0.7% of the peptides released spontaneously after 1 h from the BC-HA-MSN-SOAP dressings and a cumulative release of 30.7 ± 10.1% after 24 h. The slower release profile prolongs the antimicrobial activity of the dressings. SOAP retention can be attributed to strong electrostatic interactions between the cationic SOAP peptides and the negatively charged silanol groups of the MSNs, as well as additional interactions with the HA hydrogel matrix, which together restrict peptide diffusion. Moreover, previous work has demonstrated that SOAP release from MSNs can be blocked by capping with biomacromolecules [61].

The physical entrapment of MSNs within the HA matrix ensures uniform distribution of the carrier throughout the dressing, which can in turn improve peptide stability and release. The subsequent SOAP release from the BC-HA-MSN dressings and the kinetics profiles were fitted to different models (cf. Table S3). As shown in Fig. 5c and d, BC-HA-MSN showed an initial burst release, followed by a slow-release phase which is attributed to the diffusion of SOAP from the mesoporous channels of the MSNs [61]. The cumulative release profiles were best described by first-order kinetics, governed by diffusion and Fick's law, as evidenced by the diffusion exponent (*n* < 0.5) from the Korsmeyer-Peppas model (0.16 ≤ *n* ≤ 0.30) [[88], [89], [90]]. This indicates a diffusion-controlled process and release mechanism driven by concentration gradients. To explore the effect of proteases on the SOAP release, Coll-T1 (0.5 mg/mL) was added to the buffer. The addition of Coll-T1 resulted in a complete degradation of the grafted HA hydrogel and a concomitant release of loaded MSNs while leaving the BC intact (Fig. 5b). However, no significant effect of Coll-T1 on the SOAP release rate was observed in the dressings loaded with free SOAP micelles, due to the rapid and spontaneous release (Fig. 5c–e-g). Interestingly, a slight decrease in SOAP concentration and consequently a non-monotonic release profile was seen over time, which could be a result of HA fragments triggering peptide precipitation (Fig. 5c). By contrast, for the BC-HA-MSN-SOAP dressings, the addition of Coll-T1 resulted in a significantly faster release with 48.8 ± 5.3% of the peptides released in 1 h, plateauing at 6 h with 67.5 ± 6.5% release. This accelerated enzyme-triggered release is evidenced by an increase in the first-order kinetics constant (*k*_1_) from 0.3 ± 0.1 /h in the absence of Coll-T1 to 1.3 ± 0.1 /h in the presence of Coll-T1 for non-centrifuged samples.

To further investigate if the peptides were still trapped in the MSNs after release from the dressings, the samples were centrifuged before measuring peptide concentration in the supernatant (Fig. 5d), showing that 39.8 ± 3.0% of the SOAP was released from the MSNs after degradation of the hydrogels (Fig. 5g). The release of the fraction of SOAP still retained in the MSNs is influenced not only by concentration gradients but also by changes in colloidal stability, which may lead to particle aggregation, thereby negatively impacting on the release over time. In addition, previous work showed that a similar type of mesoporous silica (SBA-15), mediated AMP release by a concentration-driven diffusion process and subsequent MSN degradation [61]. In biological fluids, MSNs rapidly acquire a dynamic protein corona, which can retard peptide release by partially blocking the pore openings. In protease-rich environments, degradation of the protein corona can accelerate release [61]. Thus, the post-hydrogel degradation release kinetics likely reflect an interplay between peptide diffusion from MSNs, protein corona dynamics, and silica degradation, all modulated by the local wound environment.

When 1 mм of SOAP was loaded in the dressings, the concentration of free SOAP released in the buffer after 1 h was above the minimum inhibitory concentration (MIC) and the minimum bactericidal concentration (MBC) for *S. aureus* (3.1 μм) [52], both in the absence and presence of Coll-T1, for both BC-HA-SOAP and BC-HA-MSN-SOAP dressings. For the latter, Coll-T1 triggered a significantly faster and more extensive release. Overall, while SOAP release from BC-HA-SOAP dressings was driven by micelle dissociation regardless of protease presence, release from BC-HA-MSN-SOAP dressings was protease-dependent. A Coll-T1 concentration of 0.5 mg/mL, simulating high infection levels, induced a burst release, beneficial for bacteria eradication. In mildly contaminated or non-infected wounds, lower protease levels would result in slower hydrogel degradation and a gradual SOAP release, providing extended antimicrobial protection against bacterial colonization. The enzyme-responsive BC-HA-MSN-SOAP dressings enabled the AMP release to be dynamically regulated based on infection severity, where MSNs function as protective reservoirs, moderating the release even during HA enzymatic degradation, unlike non-responsive hydrogels that rely solely on AMP loading to control antimicrobial activity [91].

### 3.4 Antimicrobial activity

**Fig. 6:**
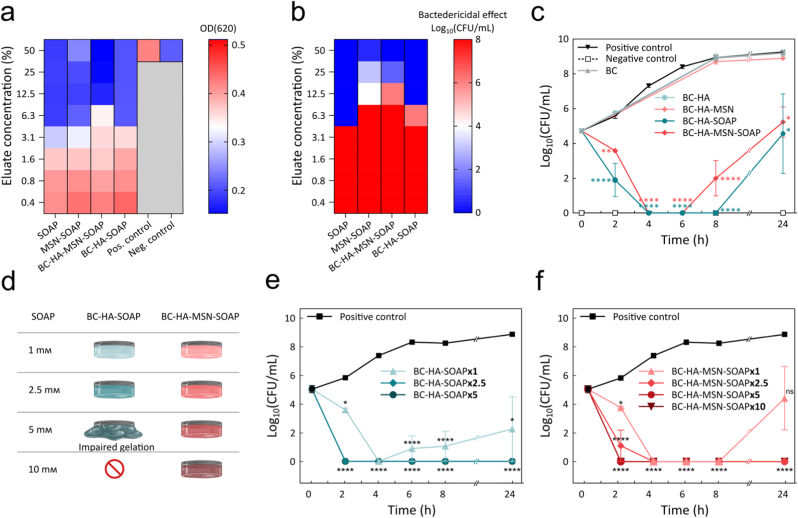
a,b) Antimicrobial activity of BC-HA±MSN-SOAP dressings and relevant controls. Broth microdilution test (*n* ≥ 6) showing a) MIC and b) MBC of eluates obtained from BC-HA-MSN-SOAP and BC-HA-SOAP dressings and respective controls. c) time-kill kinetics assay of *S. aureus* cultured with BC-HA±MSN±SOAP dressings. Statistical analysis was performed using a one-way ANOVA test by comparing the average of each sample with the positive control (*n* = 3, n.s. wherever not indicated). Multiple comparison was performed with Dunnett's post-hoc test. d) Schematic illustration of the preparation outcome for BC-HA±MSN-SOAP dressings containing 1 mм – 10 mм SOAP. e,f) Time-kill kinetics assay of *S. aureus* cultured with e) BC-HA-SOAP and f) BC-HA-MSN-SOAP dressings containing increasing SOAP concentrations. Statistical analysis was performed using a one-way ANOVA test supplemented with Dunnett's post-hoc test, by comparing the average of each sample with the positive control (n = 3).

The antimicrobial activity of the BC-HA±MSN±SOAP dressings was first evaluated using a broth microdilution test to investigate whether the SOAP peptides retained their antimicrobial activity when released. *S. aureus* was selected as the test organism, as it represents a primary pathogen in skin and wound infections [92], although SOAP peptides are also active against other clinically relevant wound pathogens, supporting their potential use for treatment of a wide range of infections [52]. BC-HA-SOAP and BC-HA-MSN-SOAP dressings were incubated in PBS for 6 h to promote SOAP release and the eluate was serially 2× diluted and combined with a suspension of *S. aureus* for 20 h (Fig. 6a and b and Figs. S12–S13, Supporting Information). Bacteria inhibition was seen up to the 4th dilution for both SOAP in solution (corresponding to 5 μм SOAP), and for the BC-HA-SOAP dressings (corresponding to 3.6 μм SOAP). BC-HA-MSN-SOAP dressings showed inhibition up to the 3rd dilution (corresponding to 2.1 μм SOAP). The interaction between the SOAP peptides and the MSNs hence resulted in a slightly lower antimicrobial activity due to the slower release, which was further confirmed by investigating MSN-SOAP not encapsulated in the dressings (Fig. 6a and b). However, these findings confirmed that the SOAP that was released from the dressings retained high antimicrobial activity. Next, we evaluated the time-dependent antimicrobial activity of the dressings using a time-kill assay (TKA). BC-HA±MSN±SOAP dressings were incubated for 24 h with approximately 5 log_10_ CFU/mL *S. aureus*, simulating the bioburden threshold that marks the transition from a colonized to an infected wound [[86], [87]]. An incubation volume of 1 mL was chosen to achieve a SOAP concentration close to the MBC, facilitating a clear distinction in antimicrobial activity between the BC-HA-MSN-SOAP and BC-HA-SOAP dressings. Bactericidal effect, typically defined as a decrease of ≥3 log_10_ CFU/mL in bacterial bioburden, was reached within 2 h by the BC-HA-SOAP dressings (1.9 log_10_ CFU/mL), and within 4 h by the BC-HA-MSN-SOAP dressings (0 log_10_ CFU/mL) (Fig. 6c), consistent with the observed *in vitro* SOAP release profile kinetics. BC-HA-SOAP dressings caused a rapid reduction in bacteria count, indicative of an instantaneous SOAP concentration close to the MBC, which produced a consistent sample variability for the 2 h timepoint and almost complete bacteria eradication at 4 h. The SOAP release from BC-HA-MSN-SOAP dressings was slower but sustained, resulting in a more gradual decrease in bacteria concentration at 2 h. Although no live bacteria could be detected after 4 h for either of the two SOAP-loaded dressings, bacteria eradication was not complete and resulted in *S. aureus* re-growth until levels around the critical concentration threshold (4.6 and 5.2 log_10_ CFU/mL for BC-HA-SOAP and BC-HA-MSN-SOAP, respectively) at endpoint under the current experimental conditions. We therefore explored the possibilities to load higher concentrations of SOAP in the dressings (Fig. 6d–Tables S4–S5, Supporting Information). Unfortunately, loading of SOAP micelles at concentrations ≥5 mм had a significant negative impact on the hydrogel cross-linking process, most likely due to the high concentration of micelles and the strong electrostatic interactions between the SOAP micelles and the HA backbone that resulted in very soft hydrogels at 5 mм SOAP and HA precipitation at 10 mм SOAP (Fig. 6d). However, in the BC-HA-MSN dressings loading of very high SOAP concentrations (10 mм) was possible without any apparent influence on the viscoelastic properties of the grafted HA hydrogels, resulting in rapid and efficient killing of the bacteria (Fig. 6e and f).

The possibilities to tailor SOAP concentrations over this wide concentration span will facilitate further optimization of the antimicrobial properties and the biocompatibility of the dressings, while alternative strategies could include combinations with conventional antibiotics [52] or inorganic agents (e.g., silver nanoparticles) to achieve complementary or additive antimicrobial effects. Although the current study focuses on preventing early infection rather than eradicating mature biofilms, future work will evaluate the efficacy of SOAP-loaded dressings against established *S. aureus* biofilms.

### 3.5 Cytocompatibility

**Fig. 7:**
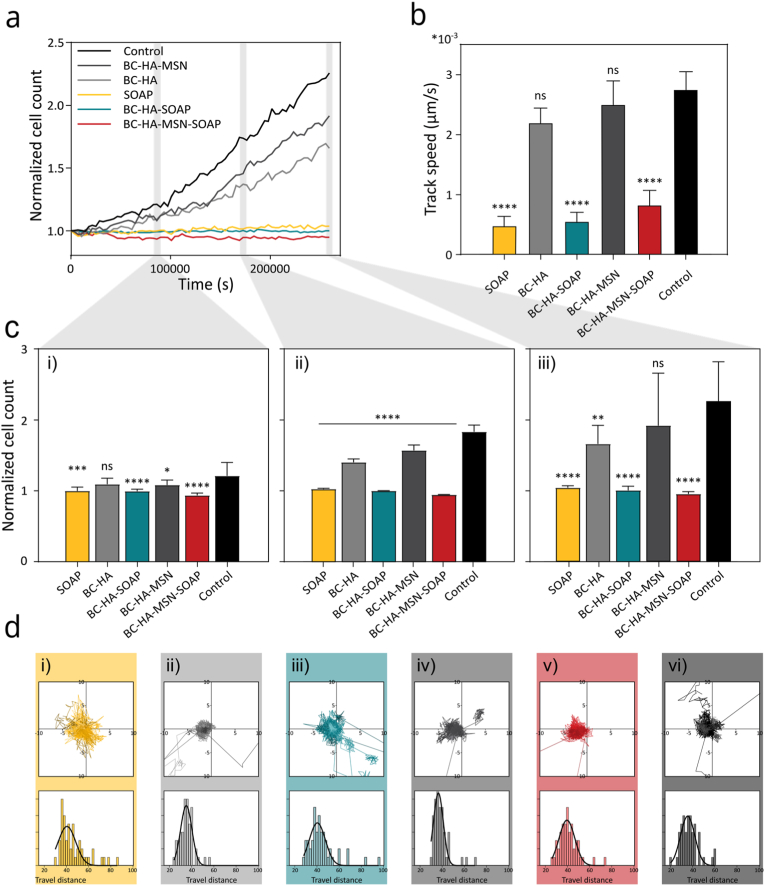
Cytocompatibility testing of wound dressings evaluated on primary human keratinocytes. a) Proliferation index (normalized to starting point) of keratinocytes cultured in medium supplemented with wound dressing eluates, evaluated for a period of 3 days. c) Normalized proliferation index at i) 24 h, ii) 48 h and iii) 72 h timepoint. b) Migration rate of keratinocytes, results displayed as mean ± standard error of the mean. Statistical analysis was performed against the control using ordinary one-way ANOVA supplemented with Dunnett's post-test (*n* ≥ 5). d) Displacement rate measured and histogram over 72 h of test for i) SOAP (simulating 100% peptide release), ii) BC-HA, iii) BC-HA-SOAP, iv) BC-HA-MSN, v) BC-HA-MSN-SOAP, vi) control sample (*n* = 50).

**Fig. 8:**
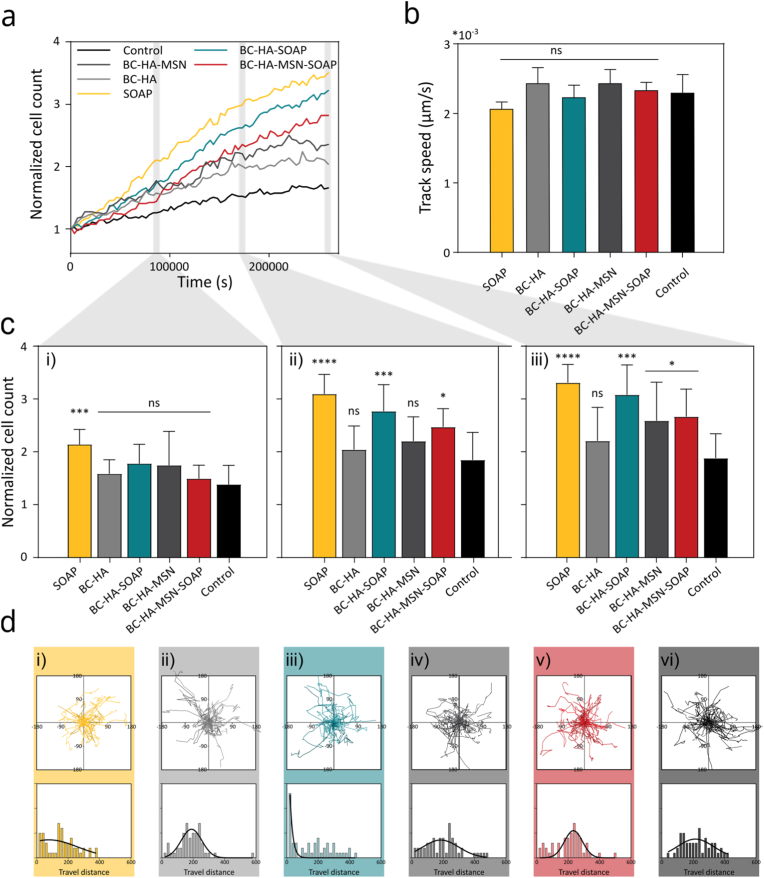
Cytocompatibility testing of wound dressings evaluated on primary human dermal fibroblasts. a) Proliferation index (normalized to starting point) of fibroblasts cultured in medium supplemented with wound dressing eluates, evaluated for a period of 3 days. c) Normalized proliferation index at i) 24 h, ii) 48 h and iii) 72 h timepoint. b) Migration rate of fibroblasts, results displayed as mean ± standard error of the mean. Statistical analysis was performed against the control using ordinary one-way ANOVA supplemented with Dunnett's post-test (*n* ≥ 6). d) Displacement rate and histogram of fibroblasts measured over 72 h of test for i) SOAP (simulating 100% peptide release), ii) BC-HA, iii) BC-HA-SOAP, iv) BC-HA-MSN, v) BC-HA-MSN-SOAP, vi) control sample (*n* = 50).

The cytocompatibility of both BC and the HA/VPM hydrogels has previously been extensively investigated. The BC dressings used here (Epiprotect™) are already used in the clinic and are an approved class IIb medical device [[93], [94]]. The HA/VPM hydrogels have been extensively investigated for biofabrication of tissue and disease models, demonstrating excellent cytocompatibility [[58], [63], [64], [65]]. However, the combination of the materials, including the SOAPs, have not previously been explored in this context. Thus, prior *in vivo* evaluation of the dressings, the cytocompatibility was carefully evaluated on human primary keratinocytes and dermal fibroblasts using a strategy based on the ISO 10993-18 standard for biological evaluation of medical devices. The dressings were incubated in 250 μL of the respective cell culture medium for 24 h. The dressings were then removed and 10% of the extractable/leachable-rich medium was supplemented to fresh cell culture medium to a final volume of 150 μL. The cells were cultured in the extractable/leachable-supplemented medium for 72 h while proliferation and migration were measured with 1 h intervals (Fig. 7, Fig. 8, and Supplementary Video 1). Keratinocytes exhibited a reduction in proliferation and migration speed when exposed to SOAP or SOAP-loaded dressings, with the most pronounced effects observed for free SOAP and the least for BC-HA-MSN-SOAP dressings, likely due to the controlled release from the MSNs. (Fig. 7a–c). Despite this reduction, SOAP-exposed keratinocytes still demonstrated increased migration activity compared to the control (Fig. 7d), suggesting that while SOAP may transiently affect proliferation rates, it does not inhibit keratinocyte motility, which is essential for re-epithelialization. In contrast, primary human dermal fibroblasts responded with a significant increase in proliferation across all conditions, with the strongest effect observed for free SOAP (Fig. 8a–c). No changes in fibroblast migration speed compared to the control were seen for any of the samples (Fig. 8b–d). Since fibroblasts play a crucial role in extracellular matrix deposition and tissue remodeling, the observed pro-proliferative effect could contribute to accelerated wound closure and enhanced tissue regeneration. While the exact mechanisms remain unclear, similar effects have been reported for other AMPs, including the full-length PLNC8 αβ peptides [95], suggesting that SOAP may interact with cellular signalling pathways involved in wound healing. The contrasting responses of keratinocytes and fibroblasts to SOAP likely arise from fundamental differences in their membrane composition and AMP-sensitive signalling mechanisms. Keratinocytes, which display a highly anionic plasma membrane, are known to respond to endogenous antimicrobial peptides such as β-defensins and LL-37 with increased migration and proliferation via EGFR-dependent STAT1/3 activation [96,97]. However, excessive exposure to cationic amphipathic peptides, such as SOAP, can amplify membrane interactions beyond physiological levels, leading to calcium stress, receptor desensitization, and transient proliferation arrest. In contrast, fibroblasts respond to cationic peptides such as LL-37 and hBD-3 by activating FGFR/JAK/STAT and GPCR/PI3K/AKT signalling pathways that promote proliferation and matrix production [98,99]. These divergent cellular responses align with normal wound-healing physiology, where keratinocytes undergo a transient stress-induced proliferative pause while fibroblasts engage in tissue-regenerative signalling. Importantly, no cytotoxic effects were observed, indicating that SOAP, when delivered in a controlled manner, remains cytocompatible. These findings highlight the potential for SOAP releasing dressings to not only control infection but also influence key cellular processes involved in wound repair. However, further investigations are needed to elucidate the molecular mechanisms underlying these effects and to optimize SOAP concentrations to balance antimicrobial activity with minimal impact on keratinocyte proliferation.

Supplementary video related to this article can be found at https://doi.org/10.1016/j.bioactmat.2026.01.042

The following is the supplementary data related to this article:

Multimedia component 2 Multimedia component 2

### 3.6 *In vivo* evaluation

**Fig. 9:**
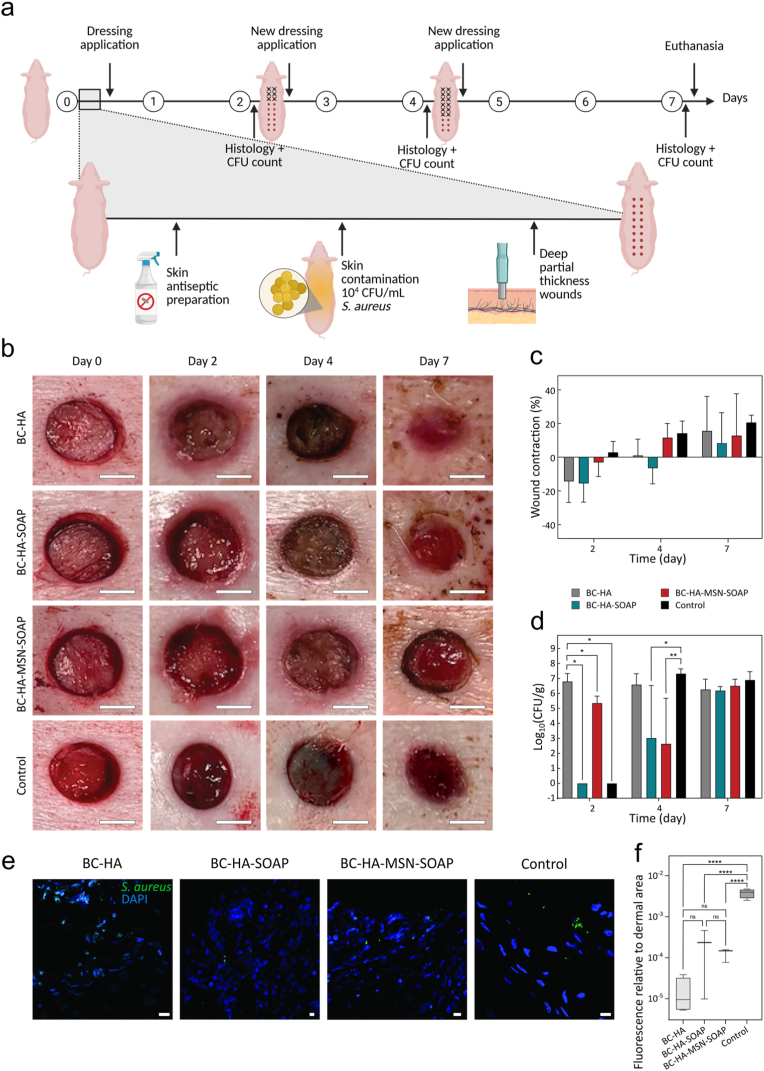
*In vivo* evaluation of antimicrobial activity. a) Schematic representation of the treatment procedure. b) Representative photos of wounds infected by *S. aureus*, scale bar: 5 mm. c) Wound contraction relative to start of experiment (day 0). d) Bacterial load in wound tissue measured by quantitative cultures, expressed as log_10_ Colony Forming Units per gram of tissue (CFU/g). Statistical analysis comparing all samples at each timepoint was performed using one-way ANOVA test supplemented with Tukey's HSD post-hoc test (*n* = 4–5). e) Immunofluorescent staining using antibodies directed against *S. aureus* (shown in green). Nuclei were counterstained using DAPI (shown in blue). Scale bar: 10 μm. f) Fluorescence quantification of *S. aureus* staining, relative to total area of the dermis. Statistical analysis was performed using ANOVA test supplemented with Holm-Sidák post-hoc test, with adjusted p < 0.05 considered different (n = 4).

To evaluate the performance of the dressings *in vivo*, we employed a porcine contamination model (Fig. 9a). The close anatomical and physiological similarities between porcine and human skin, including epidermal thickness, dermal structure, and wound healing dynamics, make this model one of the most translationally relevant preclinical models for evaluating infection management and wound healing outcomes [[100], [101]]. Additionally, this model allows for controlled bacterial contamination, closely mimicking clinical wound infections, which provides valuable insights into the efficacy of the enzyme-responsive antimicrobial dressings in a clinically relevant setting. The skin was antiseptically prepared and then contaminated with 10^4^ CFU/mL *S. aureus* (ATCC 29213), corresponding to normal flora levels, and bacteria were allowed to adhere before wound creation, thereby simulating the natural colonization and infection process of newly formed wounds by skin-resident bacteria. Thereafter, partial-thickness wounds of 10 mm in diameter were created with a biopsy punch (Fig. 9b). Three different conditions were evaluated, BC-HA, BC-HA-SOAP, BC-HA-MSN-SOAP and compared to a commercially available dressing for contaminated, colonized, and infected wounds (Sorbact® Foam) as a control. The BC-HA-SOAP, BC-HA-MSN-SOAP dressings were loaded with 1 mм SOAP. Higher concentrations of SOAP would likely exert a more pronounced antimicrobial activity but based on the *in vitro* cytotoxicity assessment (Fig. 7, Fig. 8), 1 mм was considered as a relevant starting point to balance infection control and potential impact of SOAP on wound healing.

The dressings were placed on the wounds on day 0 and replaced with new dressings on days 2 and 4. In addition to ocular inspection and measurements of wound contraction, quantitative cultures and immunofluorescent staining using antibodies directed against *S. aureus* were used to measure the changes in bacterial load in the wounds over time. Biopsies were acquired at all timepoints and subjected to histological evaluation to assess the effects on re-epithelialization. All wounds contracted progressively over the seven-day period, as shown in (Fig. 9b and c). While no significant differences in wound contraction were observed between the groups, differences in wound morphology were noted. Wounds treated with BC-HA produced more wound fluid on day 2, a more distinct scab formation on day 4, and smoother scar tissue by day 7 compared to the other groups. No significant erythema or maceration was observed for any of the conditions. Quantitative bacterial cultures on selective agar were performed on biopsies of wounds obtained from timepoints 2, 4 and 7 days. The cultures showed levels above clinical infection at day 2, corresponding to 6.48 × 10^6^ ± 3.32 CFU/g for BC-HA and 2.31 × 10^5^ ± 2.89 CFU/g for BC-HA-MSN-SOAP, expressed as geometric mean and geometric SD factor (Fig. 9d). However, the BC-HA-MSN-SOAP dressings significantly decreased bacterial levels in comparison to BC-HA (*p < 0.05*). Treatment with BC-HA-SOAP prevented the development of infection during the first two days (*p < 0.05*). A significantly reduced bacterial load in comparison to the control was evident on day 4 for wounds treated with BC-HA-SOAP (*p < 0.05*) and BC-HA-MSN-SOAP (*p < 0.01*). Interestingly, on day 4 the bacterial load in the different wounds varied substantially when treated with the SOAP loaded dressings as indicated by the large standard deviations (1.06 × 10^3^ ± 3.22 × 10^3^ CFU/g for BC-HA-SOAP and 4.36 × 10^2^ ± 1.12 × 10^3^ CFU/g for BC-HA-MSN-SOAP, expressed as geometric mean and geometric SD factor).

**Fig. 10:**
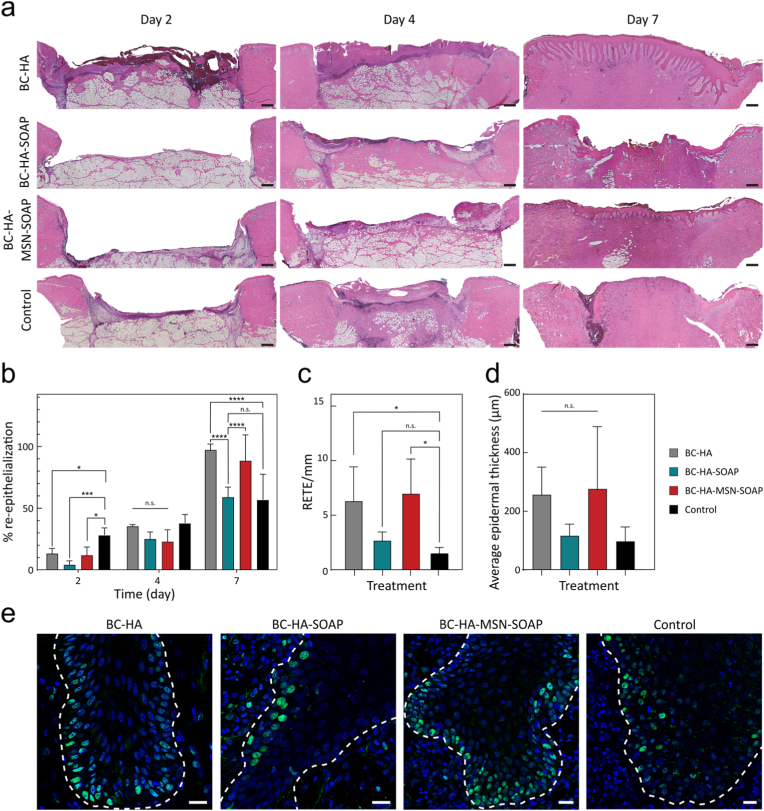
Histological evaluation of re-epithelialization and epidermal maturation *in vivo*. a) Representative Hematoxylin-Eosin (HE) stained sections of wounds from all treatment groups and timepoints. Scale bar: 250 μm b) Re-epithelialization was measured in HE stained sections and divided by total wound length, yielding percentage re-epithelialization at each timepoint. Statistical analysis comparing all samples at each timepoint was performed using two-way ANOVA test supplemented with Tukey's HSD post-hoc test (*n* = 4–5). c) The number of epidermal projections into the dermis (Rete ridges) were quantified at day 7. d) Average epidermal thickness (in μm) at day 7 was calculated by dividing total area of the neoepidemris with wound length. Statistical analysis for c) and d) was performed using one-way ANOVA tests supplemented with Tukey's HSD post-hoc tests (*n* = 4–5). e) Immunofluorescent staining of the ingrowing neoepidermis using antibodies directed against the proliferation marker PCNA (shown in green). Nuclei were counterstained using DAPI (shown in blue). White dashed lines mark the dermal-epidermal junction. Scale bar: 20 μm.

The dressings were efficiently decreasing the bacterial load in some wounds, whereas in other wounds the bacterial load remained high. Although swelling of the grafted HA hydrogel upon degradation and accumulation of wound exudate occasionally caused dressing mobility, analysis of misaligned dressings revealed no correlation with CFU count, indicating that misalignment did not contribute significantly to the observed variation. Nevertheless, careful selection of secondary dressings, adapted to each wound's characteristics and exudate volume, may contribute to maintain consistency in future studies. The heterogeneity is more likely due to intrinsic biological factors and the complexity of the wound microenvironment, with elements such as deep tissue infection, biofilm formation, pH variations, bleeding, etc. potentially further modulating AMP efficacy [102]. Rapid proliferation of *S. aureus*, with a doubling time of 20 min under ideal circumstances [103], can amplify minor differences in initial bacterial burden into substantial variations over short time frames. Individual wounds may also progress asynchronously, with bacterial reduction occurring at different times, reflecting the temporal variability of infection dynamics. Such effects warrant further studies, which could contribute to additional optimization of the dressings. The bacterial load was similar in all wounds at the end of treatment (1.87 x 10^6^ ± 4.94 CFU/g for BC-HA, 1.56 x 10^6^ ± 1.89 CFU/g for BC-HA-SOAP and 3.24 x 10^6^ ± 2.80 CFU/g for BC-HA-MSN-SOAP, expressed as geometric mean and geometric SD factor), also confirmed by the immunofluorescent staining (Fig. 9e, Fig. S14, Supporting Information). This transient effect is consistent with the release characteristics of the BC-HA-MSN-SOAP system, in which the majority of SOAP is liberated during the early, protease-rich phase of infection (Fig. 5). As inflammation subsides, protease activity and exudate volume decrease and the wound becomes progressively re-epithelialized, reducing both the stimulus for further SOAP release and the susceptibility of the wound to antimicrobial intervention. The early reduction in bacterial burden nevertheless translated into improved tissue repair, as reflected by more extensive neoepidermis and increased rete ridge formation in the SOAP-treated wounds (Fig. 10). While 1 mм SOAP was selected as a conservative starting dose to balance antimicrobial activity with cytocompatibility, the MSN platform enables substantially higher loading, which may extend antimicrobial effects beyond day 7. This is indeed possible for the BC-HA-MSN dressings which allow for an additional 10-fold increase of the loaded SOAP concentration, which can significantly facilitate further optimization of the infection management, as shown in Fig. 6f. Future dose-response studies will therefore be important for defining the optimal therapeutic window. Immunohistochemical quantification of dermal *S. aureus* showed no significant differences among treatment groups; however, all treatments exhibited significantly lower relative fluorescence than the control (p < 0.0001), indicating reduced bacterial presence and an antimicrobial effect within deeper tissue layers (Fig. 9f).

Interestingly, the release of O-HA from the dressings had a profound positive impact on neoepidermis formation (Fig. 10). Wounds treated with BC-HA and BC-HA-MSN-SOAP were significantly more re-epithelialized at day 7, compared to the control (Fig. 10b). Notably, fully re-epithelialized wounds were obtained with BC-HA treatment already after seven days, exhibiting elongated rete ridges and mature and distinctive neodermis, which is most likely an effect of the rapid HA degradation in the absence of SOAP mediated infection control (Fig. 10a). Rete ridges were significantly higher in BC-HA and BC-HA-MSN-SOAP-treated wounds, in comparison to the control (6.292 ± 3.144 Rete/mm for BC-HA, 6.965 ± 3.178 Rete/mm for BC-HA-MSN-SOAP and 1.490 ± 0.577 Rete/mm for the control, expressed as mean and SD; *p < 0.01;* Fig. 10c). Interestingly, wounds treated with BC-HA-MSN-SOAP displayed a slightly higher number of Rete ridges per millimetre compared to BC-HA (Fig. 10c). Moreover, the relative epidermal thickness was higher in the BC-HA and BC-HA-MSN-SOAP groups compared to the control (256.8 ± 94.04 for BC-HA, 276.8 ± 212.8 for BC-HA-MSN-SOAP, 116.8 ± 39.25 for BC-HA-SOAP and 97.84 ± 48.79 μm for the control, expressed as mean and SD; Fig. 10d), further highlighting the positive effects of O-HA on healing. Lastly, positive PCNA staining of cells in the ingrowing neoepidermis confirmed active proliferation (Fig. 10e). These findings align with previous literature that O-HA can favour healing[40,41,[45], [46], [47], [48]], and contradict reports suggesting that HA hydrogels alone can possess antimicrobial properties [42,43]. Moreover, the results suggest that the rapid SOAP release from the BC-HA-SOAP dressings into the wound environment could have a slightly negative effect on healing. This observation is consistent with our *in vitro* cytocompatibility data showing that SOAP reduces keratinocyte proliferation at higher concentrations, whereas migration remains unaffected. As keratinocytes are essential for re-epithelialization, controlling the early peak concentration of SOAP is important to avoid transient antiproliferative effects while still providing antimicrobial benefit. This negative effect was mitigated by loading the peptides into MSNs. The slower enzyme responsive SOAP release from the BC-HA-MSN-SOAP dressings thus managed to balance infection control during the early stages of the healing process with the release of O-HA to promote healing, resulting in lower bacterial loads and adequate re-epithelialization and epithelial maturation. Further optimization of the SOAP concentrations in the BC-HA-MSN-SOAP could enable better long-term infection management. In addition to treating infection through SOAP release and supporting wound healing via the grafted HA hydrogel, the BC dressings are designed to integrate with the scab, forming a protective barrier against bacterial infiltration. With further optimization, this approach has the potential to reduce the need for frequent dressing changes in contaminated wounds.

## 4 Conclusions

Hyaluronan-bacterial nanocellulose hybrid wound dressings with antimicrobial properties were developed by grafting an AMP-loaded enzyme-responsive hyaluronic acid hydrogel to a commercially available nanocellulose dressing (Epiprotect™). The grafted HA formed a distinct but soft hydrogel layer that was firmly anchored to the nanofibrillar BC network. HA grafting on BC had no effect on dressing conformability and the dressing retained excellent skin contact. Moreover, the dressings were relatively transparent, which facilitates ocular wound inspection without the need for dressing removal. The exudate adsorption and water retention capacity of the BC dressings were improved after HA grafting compared to BC. To grant antimicrobial properties to the dressings, a D-amino acid sequence optimized antimicrobial peptide (SOAP) was loaded in the HA hydrogel, either as micelles (BC-HA-SOAP) or loaded in an MSN carrier (BC-HA-MSN-SOAP), during the hydrogel cross-linking and grafting process. Enzyme-responsiveness was ensured using both a hyaluronidase-degradable backbone and a protease-degradable cross-linker (VPM-Az_2_). The proteolytic degradation of the dressings, which can be triggered *in vivo* by overexpression of proteases by both bacteria and the host as part of the inflammatory response, was simulated by exposing the dressings to Coll-T1. Addition of Coll-T1 resulted in complete degradation of the grafted HA hydrogel, leaving the BC intact to protect the wound from further contamination. Degradation of the grafted HA hydrogel by bacterial proteases and hyaluronidases was evaluated in an *in vitro* model, utilizing the common wound pathogen *S. aureus*. Rapid hydrogel degradation was observed when the dressings were exposed to proliferating bacteria. In contrast, negligible degradation was seen for bacterial loads below the critical infection threshold. Different SOAP release kinetics were obtained for the two different SOAP loading strategies. The BC-HA-SOAP dressings showed a burst release, whereas BC-HA-MSN-SOAP dressings showed a sustained release. For the latter, the presence of bacterial proteases effectively increased the SOAP release. The antimicrobial activity on *S. aureus* was evaluated using both a kinetic *in vitro* model, and a contaminated porcine wound model. The SOAP loaded dressings were highly antimicrobial *in vitro*, demonstrating MIC and MBC values in accordance with free SOAP. Due to the high surface area and large pore volume of the MSNs, as much as 10 mм SOAP could be loaded in the BC-HA-MSNs, further increasing the antimicrobial activity of the dressings. Both BC-HA-SOAP and BC-HA-MSN-SOAP dressings induced a decrease in proliferation of primary human keratinocytes and had a pro-proliferative effect on primary human dermal fibroblasts *in vitro*, but cells remained viable. In contaminated porcine wounds, the SOAP releasing dressings significantly reduced the bacterial load during the first 4 days. In contrast, the BC-HA dressings did not demonstrate any antimicrobial properties. Although, the antimicrobial properties of the BC-HA-SOAP were more pronounced than for BC-HA-MSN-SOAP, the positive effect on the re-epithelialization was more distinct for the latter. The BC-HA-MSN-SOAP dressings demonstrated more extensive re-epithelization compared to BC-HA-SOAP and the control and formation of a higher number of Rete ridges. The BC-HA-MSN-SOAP dressings could thus balance infection control with release of O-HA, improving the early stages of wound healing. In complex wound environments, the longevity of antimicrobial peptide activity is influenced by release dynamics and proteolytic degradation. Encapsulation of SOAP within MSNs protects the peptide from rapid enzymatic breakdown, enabling more sustained activity, and we have recently demonstrated that the D-amino acid based SOAP retains antimicrobial potency while resisting proteolytic degradation [52].

While this study demonstrates the potential of enzyme-responsive BC-HA hybrid dressings for infection-triggered AMP delivery, several challenges remain. The effectiveness of the dressing could be further enhanced by optimizing AMP loading, improving hydrogel retention on the wound, and ensuring adequate wound exudate management. The clinical translation of AMPs has historically been challenging due to instability, toxicity, and high production costs; however, the use of the D-amino acid-based SOAP, combined with localized and controlled release, mitigates some of these limitations. Further preclinical studies will focus on evaluating effects on healing outcome and biofilm disruption, as well as long-term biocompatibility, including completing the full ISO 10993 biocompatibility testing panel required for clinical translation, complementing the initial cytocompatibility and *in vivo* evaluations presented here. By integrating an already clinically used dressing material with an advanced AMP delivery system, this approach holds promise as a viable alternative for managing wound infections and reducing reliance on systemic antibiotics and new possibilities to combat the increasing problems with multidrug-resistant bacteria. Overall, the enzyme-responsive BC–HA-MSN dressing introduces a versatile strategy for localized, on-demand AMP delivery from a clinically relevant BC platform. By integrating dual enzymatic triggers with controlled peptide release, it addresses long-standing challenges in balancing infection control and tissue regeneration in advanced wound care.

## CRediT authorship contribution statement

**Elisa Zattarin:** Writing – review & editing, Writing – original draft, Methodology, Investigation, Formal analysis. **Wasihun Bekele Kebede:** Writing – review & editing, Writing – original draft, Methodology, Investigation, Formal analysis. **Zeljana Sotra:** Writing – review & editing, Writing – original draft, Methodology, Investigation, Formal analysis. **Rozalin Shamasha:** Writing – review & editing, Methodology, Investigation, Formal analysis. **Annika Starkenberg:** Writing – review & editing, Methodology, Investigation, Formal analysis. **Valentina Guerrero-Florez:** Writing – review & editing, Methodology, Investigation, Formal analysis. **Lalit Pramod Khare:** Writing – review & editing, Methodology, Investigation. **Torbjörn Bengtsson:** Writing – review & editing, Supervision, Resources, Funding acquisition, Conceptualization. **Hazem Khalaf:** Writing – review & editing, Supervision, Methodology, Investigation, Formal analysis. **Emma M. Björk:** Writing – review & editing, Supervision, Resources, Funding acquisition, Formal analysis. **Jonathan Rakar:** Writing – review & editing, Supervision, Methodology, Investigation, Formal analysis. **Johan P.E. Junker:** Writing – review & editing, Writing – original draft, Supervision, Resources, Methodology, Investigation, Funding acquisition, Formal analysis, Conceptualization. **Daniel Aili:** Writing – review & editing, Writing – original draft, Supervision, Project administration, Methodology, Funding acquisition, Formal analysis, Conceptualization.

## Data availability statement

Data will be made available on request.

## Ethics approval and consent to participate

All experiments involving human primary cells and animal models were conducted in accordance with relevant guidelines and regulations. The use of human primary cells was approved by the **Swedish Ethical Review Authority (approval no. 2018/97–31)**. All animal experiments were approved by the **Regional Animal Ethics Committee in Linköping, Sweden (permit ID 1418)**. Informed consent was obtained from all human donors or their legal representatives in accordance with institutional and national ethical standards.

## Declaration of generative AI and AI-assisted technologies in the writing process

AI tools (Grammarly and ChatGPT by OpenAI) were used for language and formatting support. The authors reviewed all content and take full responsibility for the accuracy and integrity of the final manuscript.

## Declaration of competing interest

The authors declare the following financial interests/personal relationships which may be considered as potential competing interests: Daniel Aili reports financial support was provided by Swedish Foundation for Strategic Research. Daniel Aili reports financial support was provided by Swedish Government Strategic Research Area in Materials Science on Functional Materials at Linköping University. Emma M. Björk reports financial support was provided by Swedish Research Council. Emma M. Björk reports financial support was provided by European Union. Daniel Aili reports equipment, drugs, or supplies was provided by S2Medical AB. If there are other authors, they declare that they have no known competing financial interests or personal relationships that could have appeared to influence the work reported in this paper.
