# Supplementary material for: Enzyme responsive antimicrobial hyaluronan-nanocellulose hybrid wound dressings for the treatment of infected wounds
Source: Bioact Mater. 2026 Feb 11;61:150–71. doi: 10.1016/j.bioactmat.2026.01.042 (PMC12914817; doi:10.1016/j.bioactmat.2026.01.042)
Supplement: Multimedia component 1 [file mmc1.pdf]

Supporting Information

Enzyme Responsive Antimicrobial Hyaluronan-Nanocellulose Hybrid Wound Dressings for the Treatment of Infected Wounds

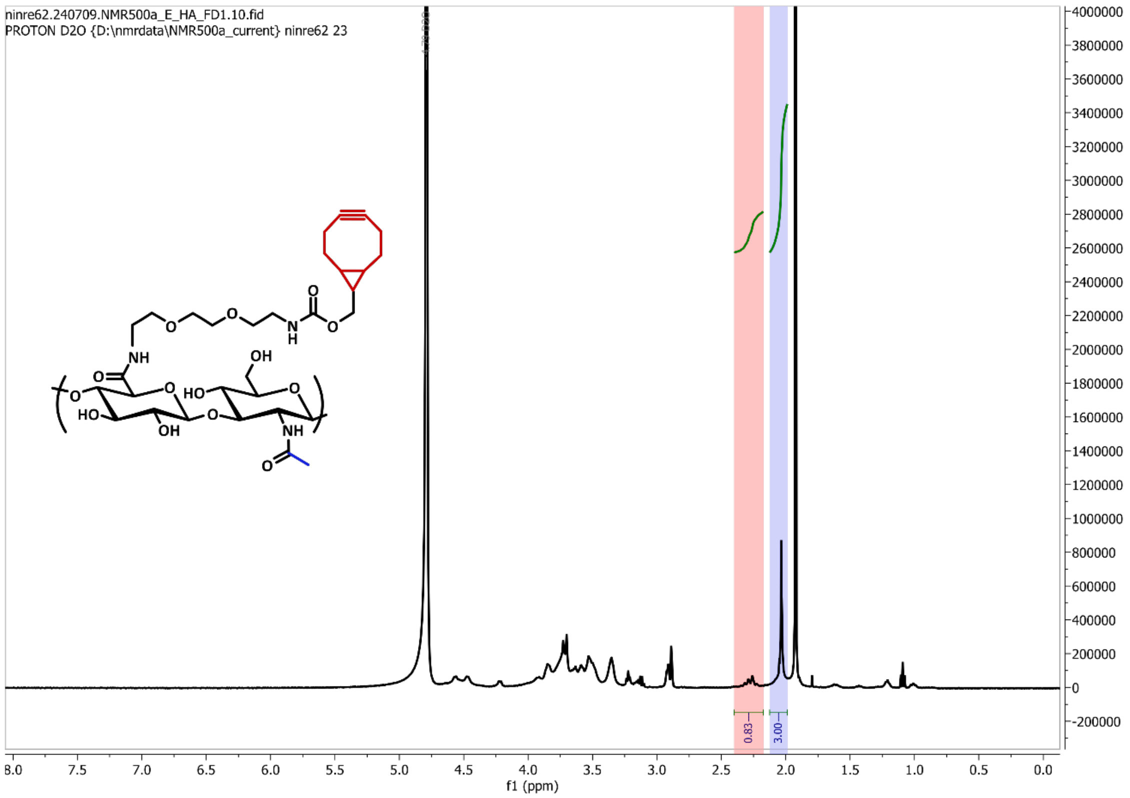

Figure S1. ^{1}H-NMR spectra of HA-BCN in D_2O indicating the degree of functionalization of HA-BCN calculated by normalizing the integral relative to the indicated BCN group with respect to the peak relative to the acetyl group in HA (1.95 ppm). A degree of functionalization of 14 % was calculated.

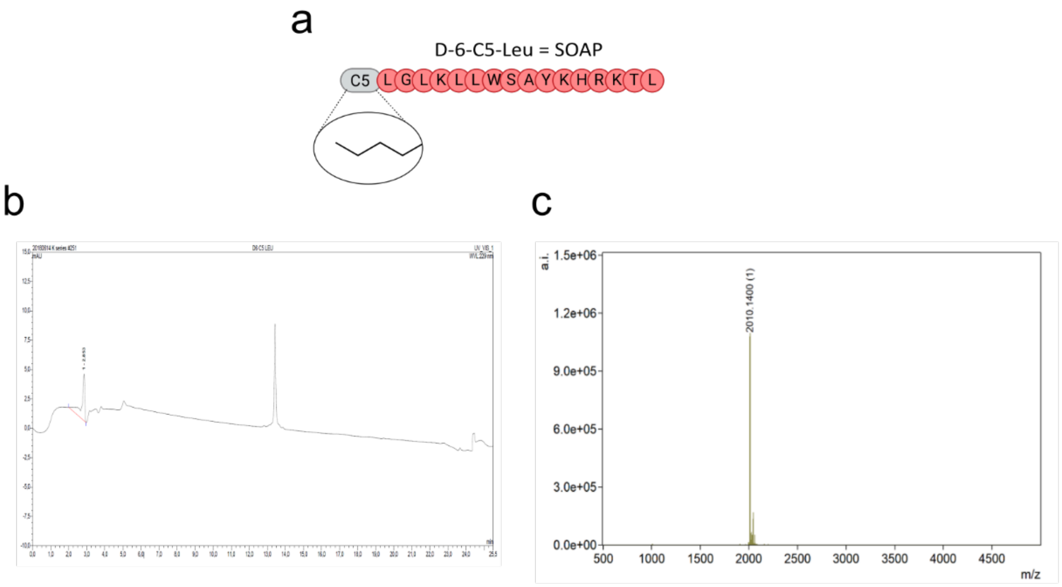

Figure S2. a) Amino acid sequence, b) HPLC chromatogram and c) MALDI-ToF mass spectra of SOAP (D-6-C5-Leu).

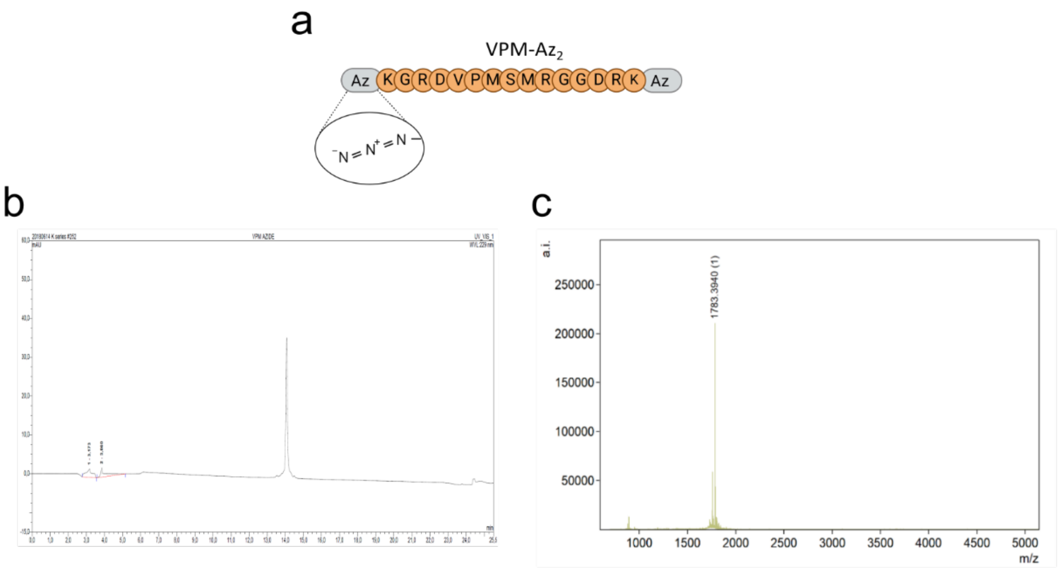

Figure S3. a) Amino acid sequence, b) HPLC chromatogram and c) MALDI-ToF mass spectra of the cross-linker VPM-Az_2.

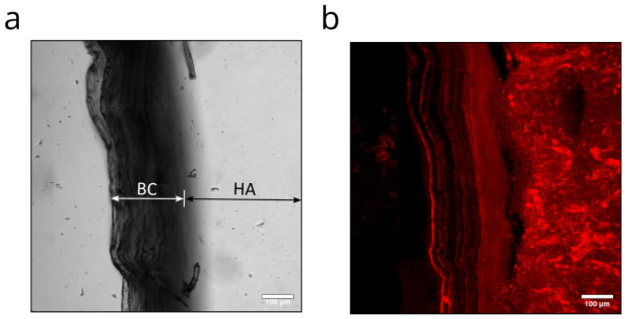

Figure S4. Confocal microscopy image of a BC-HA dressing cryosection (scale bar 100 µm).

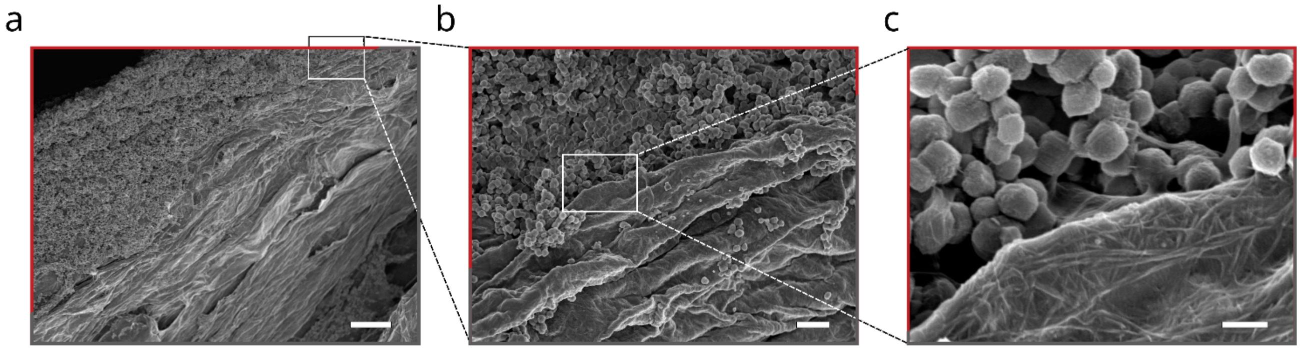

Figure S5. Scanning electron micrographs of a BC-HA-MSN dressing (cross-section) at different magnifications. Scale bar: a) 20 µm, b) 2 µm, c) 500 nm.

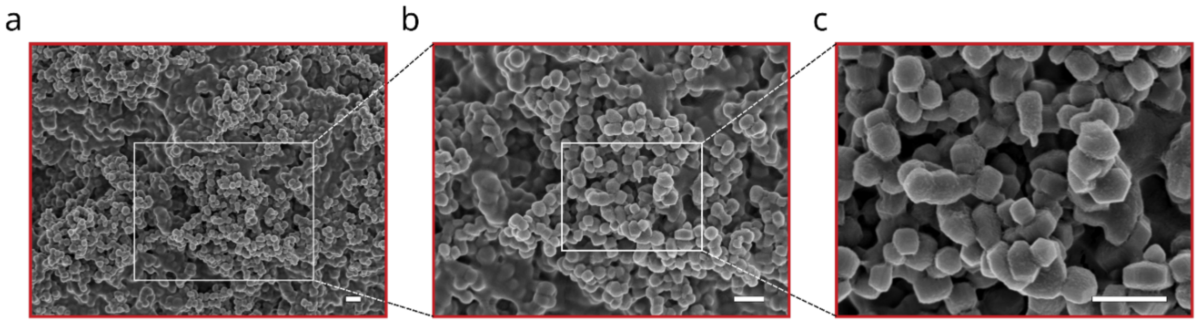

Figure S6. Scanning electron micrographs of HA-MSN dressing (top view) at different magnifications. Scale bar: a-c) 1 µm.

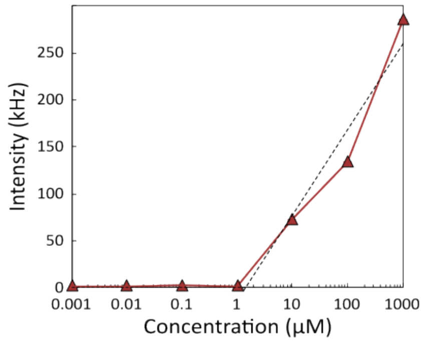

Figure S7. DLS analysis indicating the scattering intensity of 0.001 µM – 1 mM SOAP in 10 mM PBS buffer, 37 °C. Critical micelle concentration (CMC) of 1.5 µM was determined by trendline intercept.

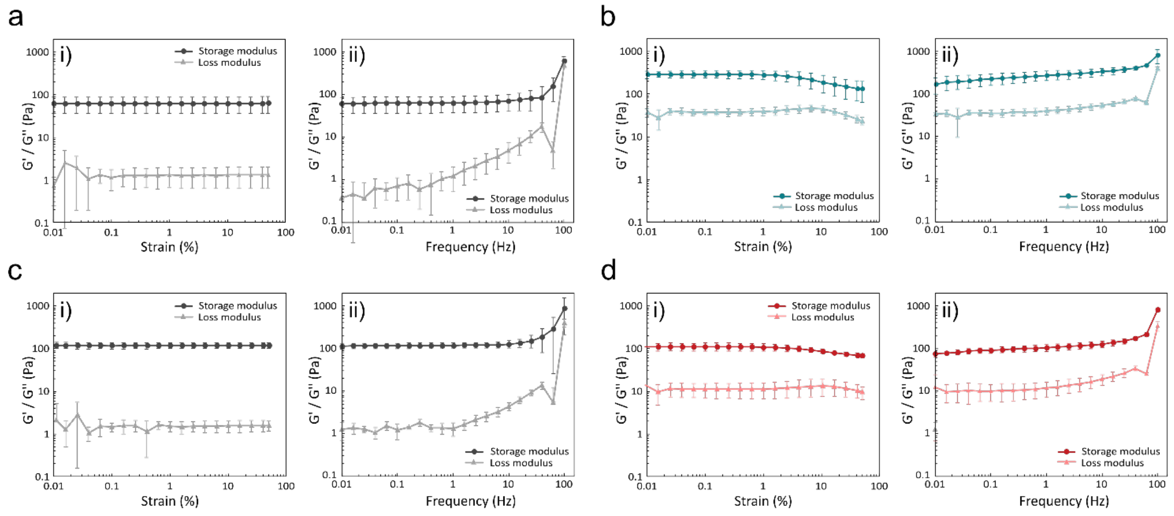

Fig S8. Storage (G') and loss (G'') modulus of a) HA and b) HA-SOAP, c) HA-MSN and d) HA-MSN-SOAP dressings evaluated by i) amplitude sweeps (1 Hz, 0.01 – 50 %) and ii) frequency sweeps (1%, 0.01-100 Hz), following a 2 h gelation (37 °C, *n*=3).

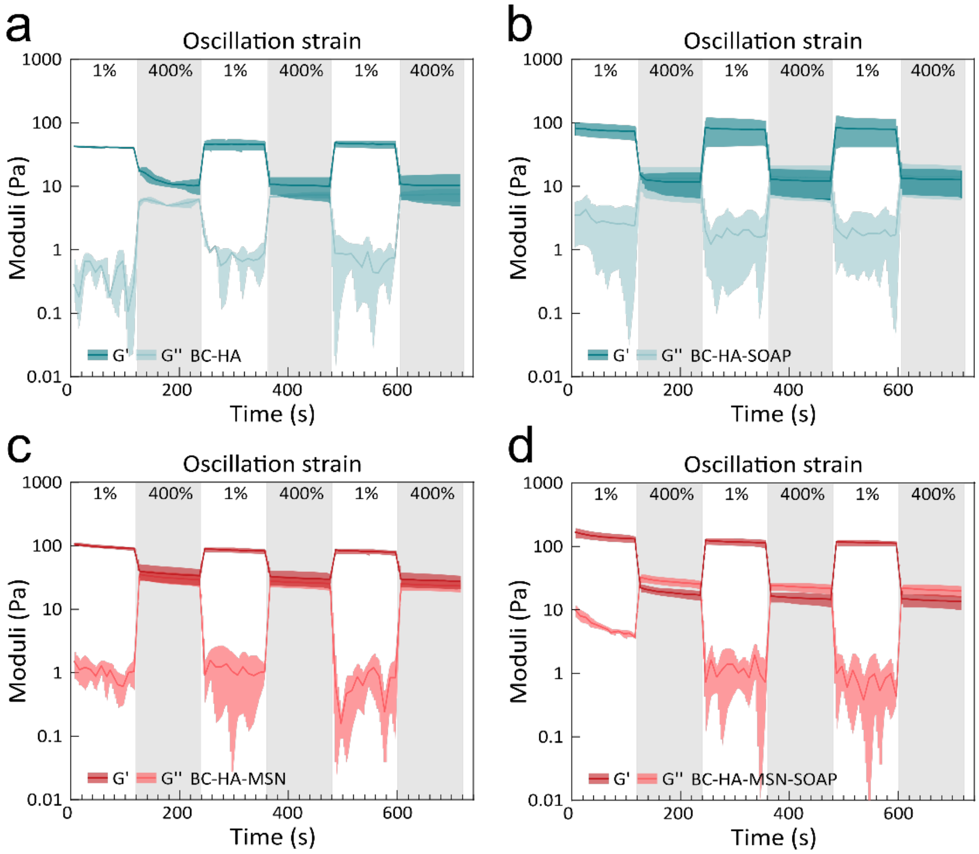

Fig S9. Strain recovery test of a) BC-HA, b) BC-HA-SOAP, c) BC-HA-MSN and d) BC-HA-MSN-SOAP dressings using alternating low-high oscillation strain cycles of 1% (non-shaded areas) and 400% (shaded areas), (1 Hz, 37 °C, *n*=3-4).

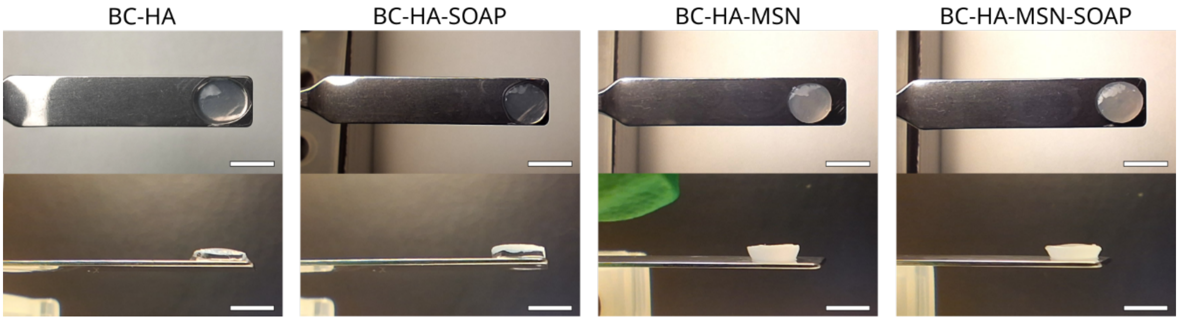

Fig S10. Long-term stability of BC-HA±SOAP and BC-HA-MSN±SOAP dressings following storage for 16 months (10 mM PBS, pH 7.4, 4 °C). Scale bar: 5 mm.

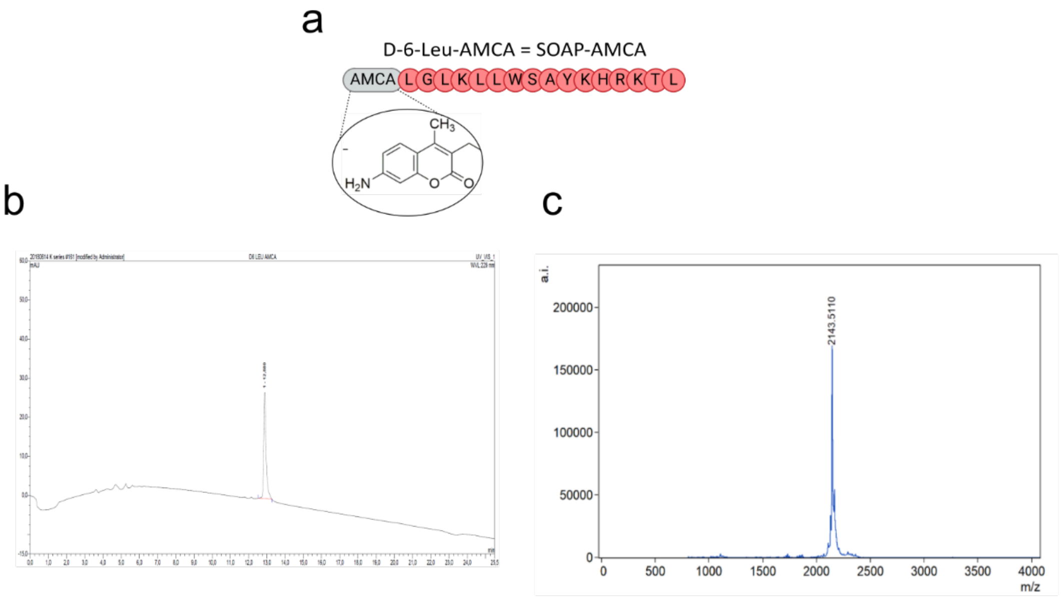

Figure S11. a) Amino acid sequence, b) HPLC chromatogram and c) MALDI-ToF mass spectra of peptide SOAP-AMCA (D-6-Leu-AMCA).

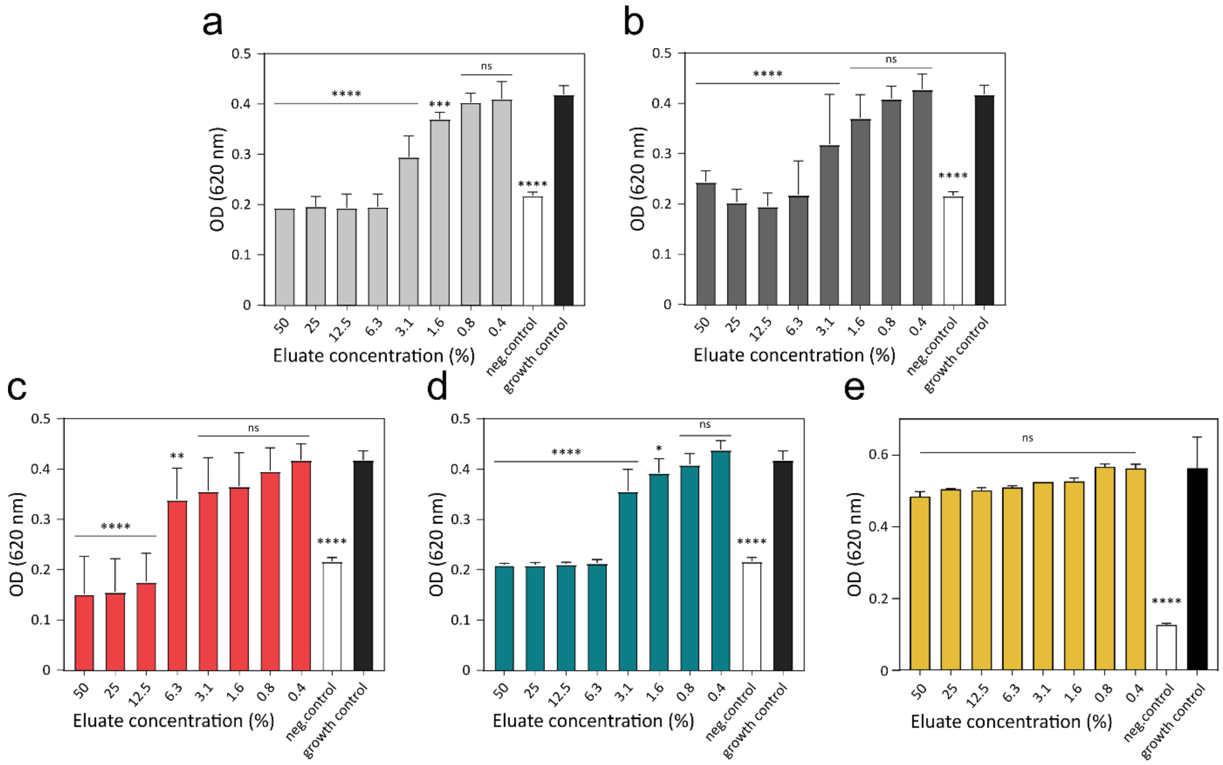

Figure S12. Antimicrobial activity evaluated by the broth microdilution method ($n \geq 6$). Graphs display the optical density reading at 620 nm at end of experiment (20 h) for the samples a) SOAP, b) MSN-SOAP, c) BC-HA-MSN-SOAP, d) BC-HA-SOAP and e) BC-HA dressings. Statistical analysis was performed with an ordinary one-way ANOVA, complemented with a Dunnett's multiple comparison test against the growth control.

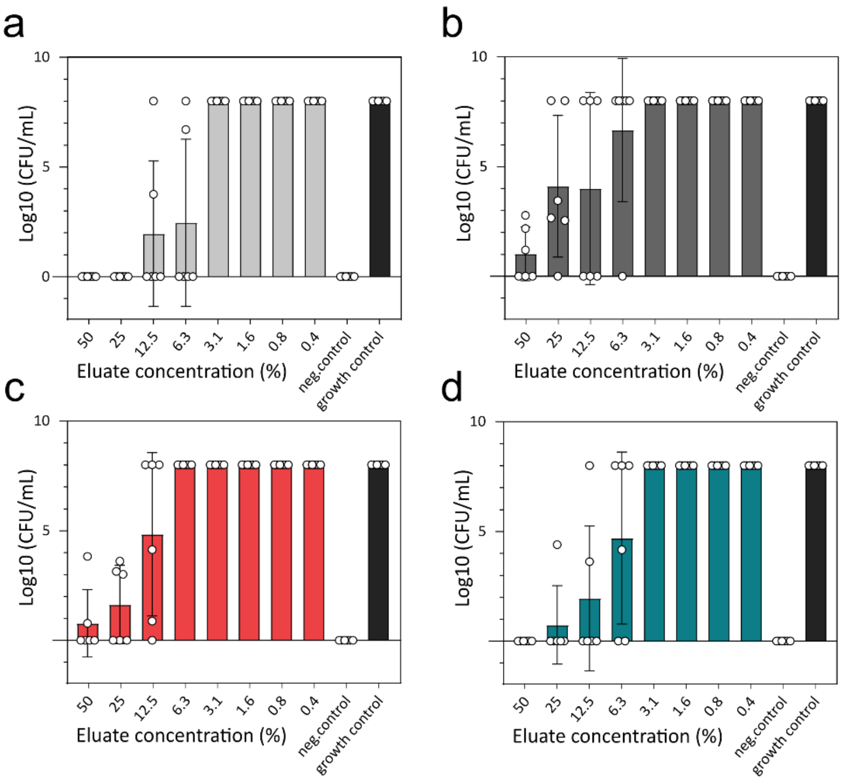

Figure S13. Antimicrobial activity evaluated by the broth microdilution method (*n* ≥ 6). Graphs display the bactericidal activity against *S. aureus* following solution plating at end of experiment (20 h) for the samples a) SOAP, b) MSN-SOAP, c) BC-HA-MSN-SOAP, d) BC-HA-SOAP dressings.

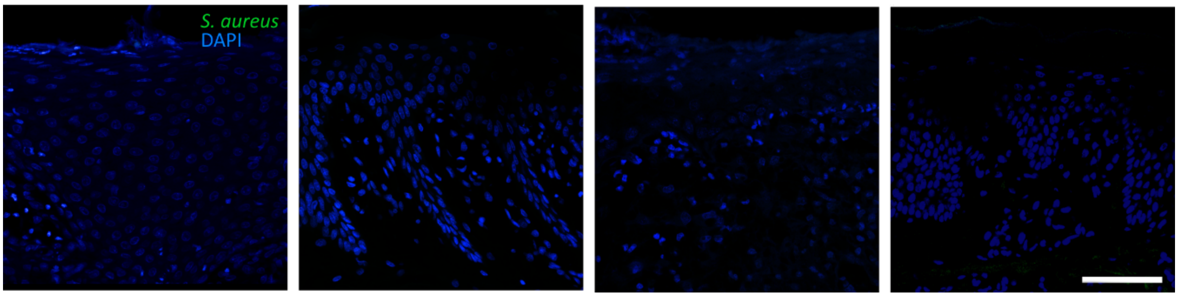

Figure S14. Negative controls of immunofluorescent staining (omission of primary antibodies) against S. aureus (shown in green) in porcine wounds. Nuclei were counterstained using DAPI (shown in blue). Scale bar: 100 µm.

Table S1. BC-HA±SOAP dressing composition and preparation procedure

Mixing order	Stock solution			BC-HA-SOAP		BC-HA	
	Component	Prepared in	Concentration	Volume (µL)	Final concentration	Volume (µL)	Final concentration
1	MQ water			3.57		5.57	
2	PBS buffer		150 mM	0.56	10 mM	0.56	10 mM
3	SOAP	MQ water	10 mM	2	1 mM	/	/
4	HA-BCN	10 mM PBS	30 mg/mL	11.67	15.71 mg/mL	11.67	15.71 mg/mL
Vortex<br>Cooling to 4 °C							
5	$VPM-Az_2$	MQ water	20 mg/mL	2.20	2.2 mg/mL	2.20	2.2 mg/mL
Vortex<br>Cross-linking at 37 °C for 2 h							

Table S2. BC-HA-MSN±SOAP dressing composition and preparation procedure

Mixing order	Stock solution			BC-HA-MSN-SOAP		BC-HA-MSN	
	Component	Prepared in	Concentration	Volume (µL)	Final concentration	Volume (µL)	Final concentration
1	SOAP	MQ water	10 mM	2	1 mM	/	/
2	MQ water			38		/	
Sonicate for 10 min						/	
3	SBA-15-OH	MQ water	35 mg/mL	5.71	10 mg/mL	5.71	10 mg/mL
Sonicate for 10 min<br>Stir for 2h at RT<br>Dry at 37 °C ON						Sonicate for 10 min	
4	PBS buffer		150 mM	0.56	10 mM	0.56	10 mM
5	MQ water			3.57		/	
6	HA-BCN	10 mM PBS	30 mg/mL	11.67	15.71 mg/mL	11.67	15.71 mg/mL
Vortex<br>Cool to 4 °C							
7	$VPM-Az_2$	MQ water	20 mg/mL	2.20	2.2 mg/mL	2.20	2.2 mg/mL
Vortex<br>Cross-link at 37 °C for 2 h							

Table S3. Kinetics parameters of SOAP release from BC-HA-SOAP and BC-HA-MSN-SOAP dressings in the presence of Coll-T1 (0.5 mg mL^{-1}, 37 °C, n=5).

System	Condition	Kinetic model							
		Zero-order				First-order		Korsmeyer-Peppas	
		1st step		2nd step					
		k_{01} (h^{-1})	R^2	k_{02} (h^{-1})	R^2	k_1 (h^{-1})	R^2	n	R^2
BC-HA-SOAP	Centrifuged	-[a]	-[a]	-[a]	-[a]	-[a]	-[a]	-[a]	-[a]
BC-HA-SOAP	Non-centrifuged	-[a]	-[a]	-[a]	-[a]	-[a]	-[a]	-[a]	-[a]
BC-HA-SOAP+Coll-T1	Centrifuged	-[a]	-[a]	-[a]	-[a]	-[a]	-[a]	-[a]	-[a]
BC-HA-SOAP+Coll-T1	Non-centrifuged	-[a]	-[a]	-[a]	-[a]	-[a]	-[a]	-[a]	-[a]
BC-HA-MSN-SOAP	Centrifuged	9.0 ± 2	0.85	0.14 ± 0.06	0.72	0.7 ± 0.2	0.93	0.16 ± 0.04	0.95
BC-HA-MSN-SOAP	Non-centrifuged	9.0 ± 3	0.76	0.8 ± 0.3	0.73	0.3 ± 0.1	0.78	0.27 ± 0.04	0.95
BC-HA-MSN-SOAP+Coll-T1	Centrifuged	20 ± 5	0.85	-[a]	-[a]	1.8 ± 0.1	0.95	0.06 ± 0.03	0.96
BC-HA-MSN-SOAP+Coll-T1	Non-centrifuged	33 ± 5	0.92	-[a]	-[a]	1.3 ± 0.1	0.99	0.30 ± 0.03	0.99

[a] non-monotonic release data. A decrease in the cumulative release percentage was observed a later time point, likely due to SOAP precipitation or re-adsorption during sampling, which resulted in non-monotonic release data. Therefore, kinetic models could not be reliably applied to this dataset.

Table S4. Composition of BC-HA-SOAP dressings containing 1 -10 mM SOAP.

Concentration in final volume	BC-HA-SOAPx1	BC-HA-SOAPx2.5	BC-HA-SOAPx5	BC-HA-SOAPx10
PBS buffer (mM)	10	10	10	
SOAP (mM)	1	2.5	5	10
HA-BCN (mg/mL)	15.71	15.71	15.71	
VPM-Az_2 (mg/mL)	2.2	2.2	2.2	
Outcome	Hydrogel	Hydrogel	Partially precipitated, impaired gelation.	Not attainable

Table S5. Composition of BC-HA-MSN-SOAP dressings containing 1 - 10 mM SOAP.

Concentration in final volume	BC-HA-MSN-SOAPx1	BC-HA-MSN-SOAPx2.5	BC-HA-MSN-SOAPx5	BC-HA- MSN-SOAPx10
PBS buffer (mM)	10	10	10	10
MSN (mg/mL)	10	10	10	10
SOAP (mM)	1	2.5	5	10
HA-BCN (mg/mL)	15.71	15.71	15.71	15.71
VPM-Az_2 (mg/mL)	2.2	2.2	2.2	2.2
Outcome	Hydrogel	Hydrogel	Hydrogel	Hydrogel
